# Functional and Structural Diversity of Bacterial Contact-Dependent Growth Inhibition Effectors

**DOI:** 10.3389/fmolb.2022.866854

**Published:** 2022-04-26

**Authors:** Bonnie J. Cuthbert, Christopher S. Hayes, Celia W. Goulding

**Affiliations:** ^1^ Molecular Biology and Biochemistry, University of California, Irvine, Irvine, CA, United States; ^2^ Molecular, Cellular and Developmental Biology, University of California, Santa Barbara, Santa Barbara, CA, United States; ^3^ Biomolecular Science and Engineering Program, University of California, Santa Barbara, Santa Barbara, CA, United States; ^4^ Pharmaceutical Sciences, University of California, Irvine, Irvine, CA, United States

**Keywords:** contact-dependent growth inhibition, toxin/immunity complex, type V secretion system, structure, toxin function

## Abstract

Bacteria live in complex communities and environments, competing for space and nutrients. Within their niche habitats, bacteria have developed various inter-bacterial mechanisms to compete and communicate. One such mechanism is contact-dependent growth inhibition (CDI). CDI is found in many Gram-negative bacteria, including several pathogens. These CDI^+^ bacteria encode a CdiB/CdiA two-partner secretion system that delivers inhibitory toxins into neighboring cells upon contact. Toxin translocation results in the growth inhibition of closely related strains and provides a competitive advantage to the CDI^+^ bacteria. CdiB, an outer-membrane protein, secretes CdiA onto the surface of the CDI^+^ bacteria. When CdiA interacts with specific target-cell receptors, CdiA delivers its C-terminal toxin region (CdiA-CT) into the target-cell. CdiA-CT toxin proteins display a diverse range of toxic functions, such as DNase, RNase, or pore-forming toxin activity. CDI^+^ bacteria also encode an immunity protein, CdiI, that specifically binds and neutralizes its cognate CdiA-CT, protecting the CDI^+^ bacteria from auto-inhibition. In Gram-negative bacteria, toxin/immunity (CdiA-CT/CdiI) pairs have highly variable sequences and functions, with over 130 predicted divergent toxin/immunity complex families. In this review, we will discuss biochemical and structural advances made in the characterization of CDI. This review will focus on the diverse array of CDI toxin/immunity complex structures together with their distinct toxin functions. Additionally, we will discuss the most recent studies on target-cell recognition and toxin entry, along with the discovery of a new member of the CDI loci. Finally, we will offer insights into how these diverse toxin/immunity complexes could be harnessed to fight human diseases.

## Introduction

In all facets of nature, living organisms exist in a variety of different environments and must adapt and compete to ensure survival. Bacteria persist in complex, mixed-species networks; to propagate they compete for space and resources with similar and disparate bacteria (Little et al., 2008; Hibbing et al., 2010; García-Bayona and Comstock, 2018). Many bacterial species employ multiple strategies to achieve a competitive advantage and to communicate with neighboring bacteria, by direct or indirect mechanisms (Cascales et al., 2007; Filloux and Sagfors, 2015; Klein et al., 2020). Direct communication involves the transmission of an effector molecule from one bacteria into a neighboring bacteria *via* direct cell-to-cell contact (Aoki et al., 2010; Russell et al., 2011; Souza DP. et al., 2015; Cao et al., 2016; García-Bayona et al., 2017; Klein et al., 2020), while indirect forms of communication involve the secretion of effector molecules into the extracellular space, which are then sensed and acquired by nearby bacteria (Corr et al., 2007; Kommineni et al., 2015; Zipperer et al., 2016; Vacheron et al., 2019).

Bacterial protein toxins are frequently employed in both direct and indirect competition mechanisms. The secretion of bacterial protein toxins into extracellular space or the direct transmission of protein toxins into neighboring cells reduces competition for space and nutrients by killing non-cognate bacterial strains (Cascales et al., 2007; Aoki et al., 2010; Filloux and Sagfors, 2015). Polymorphic toxin systems (PTS) are responsible for bacterial cytotoxic protein delivery, and require type IV (Souza D. P. et al., 2015), type V (T5SS) (Hayes et al., 2010; Aoki et al., 2011; Poole et al., 2011), type VI (Pukatzki et al., 2006; Pukatzki et al., 2007; Pukatzki et al., 2009; Hood et al., 2010; Russell et al., 2011; Souza DP. et al., 2015), and/or type VII (Bitter et al., 2009; Das et al., 2011; Sun et al., 2015; Cao et al., 2016) secretion systems to function. PTS are common in human pathogens and some have been implicated in virulence, thus understanding their mechanisms of action could be important for human health (Bitter et al., 2009; Aoki et al., 2011; Das et al., 2011; Poole et al., 2011; Sun et al., 2015). Finally, to prevent PTS auto-inhibition, delivered cytotoxic proteins are neutralized by highly specific cognate immunity proteins.

**FIGURE 1:**
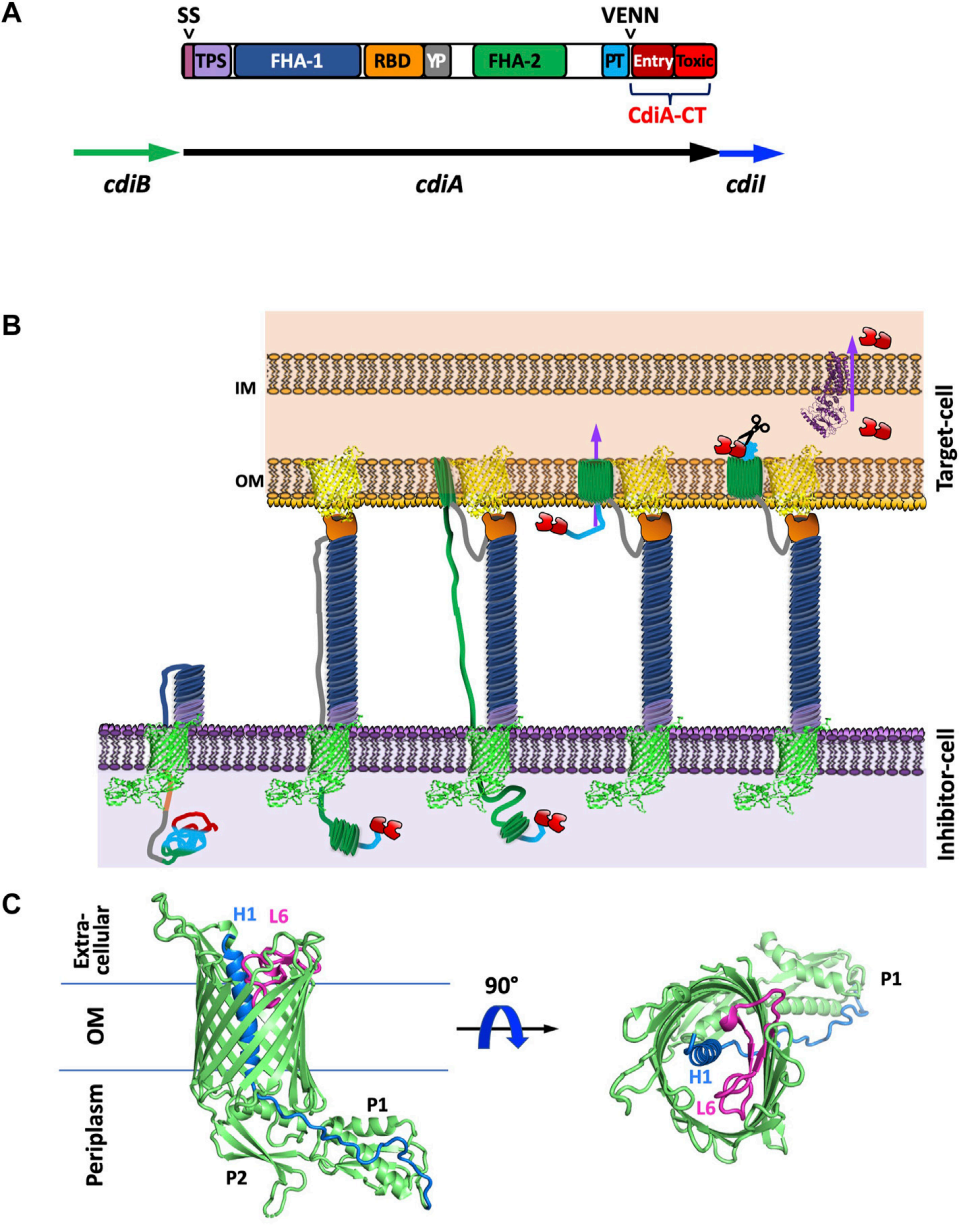
Introduction to CDI proteins and a working model of CDI toxin translocation. **(A)** Domain architecture of CdiA and organization of the *cdi* locus. CdiA is colored by domain, including the secretion signal (SS), two partner secretion (TPS) domain, FHA-1, receptor-binding domain (RBD), tyrosine-proline rich domain (YP), FHA-2, pretoxin (PT) domain, VENN motif, and CdiA-CT entry domain and CdiA-CT cytotoxic (Toxic) domain. **(B)** Schematic of CDI. CdiB (green) presents CdiA (colored by domain as in A) onto the surface of the inhibitor cell. When the CdiA-RBD (orange) recognizes its target-cell receptor (yellow), CdiA secretion continues, and FHA-2 (dark green) translocates CdiA-CT (red) into the target-cell. Once in the target-cell periplasm, the CdiA-CT is cleaved after the PT-motif, the entry domain recognizes a specific inner membrane receptor (purple) and the cytotoxic domain is translocated across the inner membrane into the cytosol. **(C)** Structure of CdiB. CdiB is an OMP β-barrel. Key components like α-helix H1 (blue) and loop L6 (pink) are highlighted [PDB ID 6WIL (Guerin et al., 2020)].

Arguably, the best characterized PTS are colicins and S-type pyocins, produced by *Escherichia coli* and *Pseudomonas* species, respectively (Cascales et al., 2007). These PTS utilize indirect forms of competition, where the secreted toxin is taken up by neighboring bacteria. Colicins are small cytotoxic proteins utilized by enteric bacteria to specifically kill bacteria of the same or closely related species (Gordon and O'Brien, 2006; Cascales et al., 2007; Ghequire and De Mot, 2014). To prevent autoinhibition, colicin-producing bacteria also express a cognate immunity protein to inactivate its cognate colicin (Graille et al., 2004; Yajima et al., 2004; Gordon and O'Brien, 2006; Cascales et al., 2007; Papadakos et al., 2012). Colicins, like many PTS toxins, are modular proteins consisting of multiple domains; a conserved N-terminal translocation domain, a central receptor-binding domain, and a polymorphic C-terminal cytotoxic domain responsible for cell death (Figure 1A **)**. *E. coli* produces several different colicin effector proteins with a diverse range of toxic activities. A subset of colicins have rRNase (e.g., ColE3) or tRNase (e.g., ColE5 and ColD) activity, while other colicins have DNase activity or form pores in phospholipid bilayers (Cascales et al., 2007). Following the discovery of colicins and other bacteriocins in the mid 20th century, other PTS involved in bacterial survival and pathogenesis have been identified, one such mechanism is contact-dependent growth inhibition (CDI).

In Gram-negative bacteria, CDI is a widespread inter-bacterial competition mechanism (Aoki et al., 2010). CDI proteins have also been implicated in contact-dependent signaling (CDS), indicating that CDI fulfills roles beyond competition (Garcia et al., 2013; Garcia et al., 2016; Danka et al., 2017; Garcia, 2018; Ocasio and Cotter, 2019). CDI involves a two-partner secretion system (TPS or T5SS), wherein the TpsB protein, CdiB, a β-barrel outer membrane protein (OMP) exports and displays the large TpsA protein, CdiA, on the cell surface **(** Figure 1B **)**. Like colicins, CdiA is highly modular, and possesses a cytotoxic C-terminal region (CdiA-CT) **(** Figure 1A **)**. When CdiA interacts with specific outer membrane target-cell receptors, the cytotoxic CdiA-CT region is cleaved and translocated into the cytoplasm of the target bacterium where its cytotoxic activity results in growth inhibition and cell death (Aoki et al., 2005; Aoki et al., 2010). While only a small subset of CdiA-CT toxins have been experimentally characterized, most are nucleases (Poole et al., 2011; Morse et al., 2012; Beck et al., 2014; Morse et al., 2015; Johnson et al., 2016a; Johnson et al., 2016b; Batot et al., 2017; Jones et al., 2017; Michalska et al., 2017; Gucinski et al., 2019; Allen et al., 2020), and one possesses pore-forming activity (Aoki et al., 2010). To prevent susceptibility to their own toxins, CDI^+^ bacteria express a cognate immunity protein, CdiI, to specifically bind and inactivate CdiA-CT toxicity.

A number of CDI toxin/immunity protein complexes from diverse organisms have been structurally characterized: *Burkholderia* species (Morse et al., 2012; Johnson et al., 2016b)*, Enterobacter cloacae* (Beck et al., 2014), *E. coli* species (Morse et al., 2012; Johnson et al., 2016a; Jones et al., 2017; Michalska et al., 2018; Gucinski et al., 2019), *Klebsiella pneumoniae* 342 (Gucinski et al., 2019), *Pseudomonas aeruginosa* PABL017 (Allen et al., 2020) and *Yersinia* species (Morse et al., 2015; Batot et al., 2017). While the toxin and immunity proteins are incredibly diverse in protein sequence, the majority of the known CDI toxin structures can be placed into two protein superfamilies: BECR (Barnase/EndoU/Colicin/RelE) (Beck et al., 2014; Michalska et al., 2017; Michalska et al., 2018; Gucinski et al., 2019; Allen et al., 2020), and PD-(D/E)XK (Morse et al., 2012; Morse et al., 2015; Johnson et al., 2016b; Gucinski et al., 2019) (Table 1). Only two characterized CDI toxins are placed outside of these superfamilies, one of which is the first characterized bacterial RNase A superfamily member (Batot et al., 2017) and the other does not belong to any previously characterized protein family, but is designated as an Ntox28 toxin (Johnson et al., 2016a). Here, we will review the current information on CDI systems with a focus on a structural and functional understanding of CDI toxin/immunity protein complexes. We will conclude with a discussion of potential applications for CDI toxin/immunity protein complexes in therapeutics and biotechnology.

**TABLE 1:** Summary of different CDI toxin activities, active sites, and homology to other known structures.

CdiA-CT Organized by Family	Non-CDI CdiA-CT homologs	CDI CdiA-CT homologs	Activity or substrate	Co-Factors	Active site residues	CdiA-CT structure	CdiI structure	CdiA-CT Buried Surface Area at the CdiA-CT/CdiI Interface	CdiA-CT/CdiI Interactions
**BECR proteins**									
EC3006 (PDB ID 6CP8)	ColD (3.6 rmsd, 3% ID)	Kp342 (2.8 rmsd, 15% ID)	uncharged tRNA_GAU_^Ile^		**K204**, Y208, H256, **R260**, T330	βββαααααβββ	αααααααααα	1928 Å^2^; 20.4%	8 salt bridges
									23 H-bonds
Kp342 (PDB ID 6CP9)	ColD (3.5 rmsd, 8% ID)	EC3006	uncharged tRNA_GAU_^Ile^	GTP, EF-Ts, EF-Tu	**K157**, Y160, **H170, R252**, T255	αααααββββ	αβββββββββ	939 Å^2^; 13.2%	5 salt bridges
	BrnT (2.5 rmsd, 9% ID)	NC101 (3.2 rmsd; 7% ID)							15 H-bonds
	Colicin E5 (3.0 rmsd, 7% ID)	PABL017 (3.8 rmsd, 10% ID)							
		PABL017 (3.8 rmsd, 10% ID)							
		Ykris (4.3 rmsd, 7% ID)							
NC101 (PDB ID 5I4Q/5I4R)	BrnT (2.4 rmsd, 12% ID)		tRNA acceptor stem	GTP, EF-Ts, EF-Tu	**His248, Arg200, Glu236**	ββββααββββ	αααα	775 Å^2^; 13.9%	3 salt bridges
	ColE5 (2.5 rmsd; 15% ID)	Kp342 (2.9 rmsd, 7% ID)							8 H-bonds (5I4Q)
	ParE (3.4 rmsd, 13% ID	EC3006 (3.5 rmsd, 5% ID)							
STECO31 (PDB ID 5HKQ)	Nsp15 (3.5 rmsd, 10% ID)	Ykris (3.3 rmsd, 5% ID)	tRNA_UUC_^Glu^		**H187, H204, K261**, T262, H321	ββαββββαβββ	ααββαβαβαα	1,665 Å^2^; 20.0%	8 salt bridges
	EndoU (3.3 rmsd, 11%ID)	EC3006 (3.2 rmsd, 5%ID)							23 H-bonds
		Kp342 (3.8 rmsd, 7%ID)							
ECL (PDB ID 4NTQ)	ColE3 (2.5 rmsd, 17% ID)	N/A	16S rRNA		D203, **D205**, H207, K214	αββββββ	ββββαβαββαβα	1,396 Å^2^; 27.7%	16 H-bonds
PABL017 (PDB ID 6D7Y)	ColD (2.4 rmsd, 10%ID)	Kp342 (3.8 rmsd, 10% ID)	tRNA^Gln^, tRNA^Pro^		**H3372**	ββαααββββ	βαβαβββαβαββ	1,345 Å^2^; 23.0%	6 salt bridges
	BrnT (2.8 rmsd, 10% ID)	NC101 (3.2 rmsd, 13% ID)							11 H-bonds
	RelE (3.4 rmsd, 7% ID)	Ykris (3.3 rmsd, 9% ID)							
**PD-(D/E)XK**									
TA271 (PDB ID 4G6U)	Vsr endonuclease (3.1 rmsd, 6.1% ID)	YpIII (1.7 rmsd, 64% ID)	DNase	Zn^2+^	E177, D198, S209, K211	αααααβαααβββααββαβαβ	ββββααααββββαβα	1,170 Å^2^; 10.2%	5 salt bridges
	Endonuclease (3.5 rmsd, 7.7% ID)	BpE479 (3.5 rmsd, 11% ID), Bp1026b (4.4 rmsd, 9% ID), EC3006 (6.1 rmsd, 5% ID)							15 H-bonds
YpIII (PDB ID 4ZQU)	Vsr endonuclease (3.1 rmsd, 6.7% ID)	TA217 (1.7 rmsd, 64% ID)Bp1026b (3.8 rmsd, 12% ID)	DNase	Zn^2+^	E177, D198, S209, K211	αβββααβββαβ	ββββαββαααβββββα	1,122 Å^2^; 12.0%	3 salt bridges
	Endonuclease (3.4 rmsd, 8.1% ID)	BpE479 (3.5 rmsd, 10% ID)							14 H-bonds
Bp1026b (PDB ID 4G6V)	MspJI restriction endonuclease (2.2 rmsd, 14% ID)	BpE479 (2.9 rmsd, 19% ID)YpIII (3.8 rmsd, 12% ID)	tRNA_1B_^Ala^	Mg^2+^	**E187, D214**, D223, **K242**	βαβββαβαββ	βααββαβαββ	1,062 Å^2^; 14.9%	5 salt bridges
	Xylose-like endonuclease (3.3 rmsd, 11% ID)	TA217 (4.4 rmsd, 9.2% ID)							15 H-bonds
BpE479 (PDB ID 5J4A)	MspJI restriction endonuclease (2.8 rmsd, 10.5% ID)	Bp102b (2.9 rmsd, 19% ID)YpIII (3.5 rmsd, 10% ID)	tRNase	Mg^2+^	**E204, D229**, D243, **H275**, D285	αβββαβαααβ	βββααβααα	962 Å2; 14.2%	2 salt bridges, 10 H-bonds
	Bse634I restriction endonuclease (3.3 rmsd, 10% ID)	TA217 (3.5 rmsd, 11% ID)								
	Xylose-like endonuclease (3.4 rmsd, 13.8% ID)									
**RNase A/BECR**									
Ykris (PDB ID 5E3E)	Angionenin (3.4 rmsd, 4% ID)	STECO31 (3.8 rmsd, 5% ID)	RNase/cCMP hydrolysis		**H175, T276, Y278**, R186	ααβααβββββ	αααααααα	1,019 Å^2^; 15.1%	5 salt bridges, 19 H-bonds
	Pancreatic RNase (3.2 rmsd, 5% ID)	PABL017 (4.3 rmsd, 11% ID)								
	RNase ZF-1a (3.2 rmsd, 6% ID)	EC3006 (3.8 rmsd, 8% ID), Kps342 (4.2 rmsd, 7% ID)								
	RelE (3.6 rmsd, 11% ID)								
**Uncharacterized**									
UPEC536 (PDB ID 5J5V/5J43)	N/A	N/A	tRNase	CysK	D155, **W176, H178, E181**	ααββαα	αααααααααα	1,062 Å^2^; 16.2%	2 salt bridges, 12 H-bonds

Homology is described by root mean square deviation (rmsd) and sequence identity (ID), not applicable (N/A) is given when there are no known homologs. Secondary structure is provided for toxin and immunity proteins (α for α-helix, β for β-strand), as well as a description of interactions and toxin buried surface area (Å^2^ and %) at the toxin/immunity interface. Toxin active site residues involved in the toxin/immunity interface are bolded. Toxin/immunity interface areas were determined by PISA (Krissinel and Henrick, 2007), and the interactions were determined by pdbSUM (Laskowski et al., 2018).

## Mechanisms of CDI

The CDI pathway is a widespread inter-bacterial competition mechanism present in α, β, and γ-proteobacteria (Aoki et al., 2010). In many Gram-negative species, CDI is encoded by three genes, *cdiB, cdiA,* and *cdiI,* from a *cdiBAI* gene cluster (Figure 1A **)**. CdiB and CdiA form a TPS system by which the β-barrel OMP CdiB exports and assembles CdiA on the cell surface. CdiA is a large protein that consists primarily of a scaffold formed by filamentous hemagglutinin adhesin (FHA) repeats, which extends several hundred angstroms from the cell surface (Aoki et al., 2010; Hayes et al., 2010). At the C-terminus of CdiA is the functional toxin region (CdiA-CT) (Figure 1A).

The mechanism involved in CDI toxin delivery can be broadly defined by four steps: target-cell recognition and binding, outer membrane translocation, toxin cleavage and inner membrane translocation into the cytosol, and growth inhibition (Figure 1B **)**. When CdiA contacts specific outer membrane receptors on neighboring bacteria, CdiA-CT is cleaved away from the CdiA N-terminal region and translocated into the neighboring cell’s cytosol. If the neighboring bacteria is a non-isogenic target-cell, CdiA-CT acts as a toxin to inhibit target-cell growth (Aoki et al., 2005; Aoki et al., 2010). If the neighboring cell is an isogenic CDI^+^ cell, it will express an immunity protein (CdiI) that specifically binds and inactivates its cognate CdiA-CT. Thus, CdiI inactivation of CdiA-CT in neighboring isogenic cells allows for continued cell growth; while neighboring non-isogenic cells are not protected and are thus susceptible to CDI. The interaction between cognate CdiA-CT and CdiI is highly specific with nanomolar binding affinity (Morse et al., 2012; Nikolakakis et al., 2012; Beck et al., 2014).

CDI^+^ cells only target closely related bacterial strains. Historically, the clearest benefit of CDI to CDI^+^ bacteria is the competitive growth advantage it gains over non-CDI bacteria, wherein CDI^+^ bacteria survive CDI and have increased access to resources in the depleted bacterial pool. However, with the emergence of CDS research (*Genetics of CDI and Contact-Dependent Signaling*), CDI^+^ bacteria likely can also use CDI machinery to communicate with nearby isogenic and other closely related bacteria (Garcia et al., 2013; Garcia et al., 2016; Danka et al., 2017; Garcia, 2018; Ocasio and Cotter, 2019).

### CdiA is a Modular Protein

While CdiA proteins vary in size (180–630 kDa), they all share the same overall architecture (Figure 1A). CdiA proteins display high conservation at the large N-terminal region which encompasses an N-terminal TPS transport domain, and two large filamentous repeat regions, FHA-1 and FHA-2. Sandwiched between the FHA-1 and FHA-2 regions are two additional domains: the receptor-binding domain (RBD) and the YP domain—a tyrosine and proline enriched region. CdiA FHA regions form extended β-helices resulting in a large rod-like structure that protrudes from the cell-surface and presents the RBD at its tip (Aoki et al., 2010; Hayes et al., 2010). While both the YP and RBD domain are required for target-cell adhesion, the RBD is responsible for contact with target-cell receptor proteins and the YP domain is likely responsible for CdiA secretion arrest (described in *Current Understanding of CDI Toxin Delivery*) (Ruhe et al., 2018). After the FHA-2 region is the pretoxin (PT or CdiA-PT) domain that terminates with a highly conserved PT motif: a VENN motif in most species, an (E/Q)LYN motif in *Burkholderia* species (Nikolakakis et al., 2012), one of five distinct motifs in *P. aeruginosa* (WVHN, TENN, LYVT, DAMV, NEALV) (Mercy et al., 2016; Allen and Hauser, 2019), or an LPEN motif in some classes of *Acinetobacter* species (De Gregorio et al., 2019). The PT-motif demarcates the beginning of the highly polymorphic CdiA-CT region which is typically 200–450 residues. In general, CdiA-CT encompasses an N-terminal cytoplasmic entry domain and a C-terminal cytotoxic domain.

CdiA is highly modular: individual domains control distinct CDI mechanisms. Single, independently functioning domains are responsible for interactions with the target cell, entry into the target-cell cytosol, and cytotoxicity. For example, in *P. aeruginosa* distinct RBDs, CdiA-CT entry and cytotoxic domains have been identified; various arrangements of these modules in CdiA proteins result in altered target-cell entry and toxicity mechanisms (Allen and Hauser, 2019). As each of these modules act independently, functional chimeric CdiA proteins can be created to control target-cell recognition, entry mechanism, or toxin activity (Aoki et al., 2011; Nikolakakis et al., 2012; Ruhe et al., 2013). One such example of a CdiA chimera protein is generated by introducing a non-native CdiA-CT region directly following the species-specific PT-motif and results in the exchange of entire toxin activities between diverse CDI^+^ species. These chimeric CdiA proteins retain the ability to deliver non-cognate toxins into the target-cell, resultantly only cells expressing the cognate immunity protein are resistant to growth inhibition by the chimeric CdiA (Aoki et al., 2011; Nikolakakis et al., 2012; Ruhe et al., 2013). The modularity of the cytotoxic CdiA-CT region allows for the exchange and acquisition of new toxins through horizontal gene transfer (HGT - further discussed in *Genetics of CDI and Contact-Dependent Signaling*) and recombination inside and outside of the *cdi* loci. As different classes of secreted effectors possess toxins with homology to known CdiA-CT cytotoxic domains, there is substantial evidence for such genetic transfer (Poole et al., 2011; Mercy et al., 2016; Ruhe et al., 2016; Jones et al., 2017; Allen and Hauser, 2019).

Further, the CdiA-CT region is composed of two modular domains; the CdiA-CT entry domain plays a critical role in toxin translocation (Willett et al., 2015a), while the CdiA-CT cytotoxic domain is responsible for cytotoxic activity (Figure 1A). The CdiA-CT entry domains recognize specific inner membrane proteins (IMPs) to mediate toxin transport across the inner membrane (Willett et al., 2015a). CdiA-CT regions with different toxic activity but conserved entry domains rely on the same translocation mechanism to gain entrance into the cytosol. Thus, when fusion proteins are generated that exchange the CdiA-CT entry domains between CdiA-CT regions, the mechanism of target-cell inner membrane translocation is dictated by its entry domain. For example, expression of the *Burkholderia pseudomallei* 1026b specific IMP was required for the entry of an *E. coli* CdiA fusion containing the full *B. pseudomallei* CdiA-CT region into *E. coli* cells (Willett et al., 2015a), demonstrating the requirement for specific IMPs for CdiA-CT target-cell entry amongst different bacterial species.

### Target-Cell Recognition by the CdiA Receptor-Binding Domain

When inhibitor-cell CdiA makes contact with a specific target-cell outer membrane receptor, the cytotoxic CdiA-CT region is cleaved and translocated into the cytoplasm of the target bacterium by a multi-step, partially characterized mechanism (Aoki et al., 2005; Aoki et al., 2010), Figure 1B. Different CDI systems recognize distinct target-cell receptors. Class I *E. coli* CdiA proteins, like the well-characterized CdiA^EC93^, bind to OMP BamA (Aoki et al., 2008; Ruhe et al., 2013), Class II *E. coli* CdiA proteins (e.g., CdiA^EC536^) recognize OmpF/OmpC heterotrimers (Beck et al., 2016), and Class III *E. coli* CdiA proteins (e.g., CdiA^STEC031^) recognize Tsx (superscripts indicate the shortened names of the bacterial strain) (Ruhe et al., 2017). While the majority of currently identified target-cell receptors are OMPs, Class VI *E. coli* CdiA (e.g., CdiA_2_^STECO31^ or CdiA^STEC4^) and *B. pseudomallei* 1026b CdiA_II_ recognize a glycolipid, lipopolysaccharide (LPS) as their target-cell receptor (Koskiniemi et al., 2015; Halvorsen et al., 2021); notably, the *E. coli* STEC_O31 CDI operon has an additional *cdiC* gene that appends a lipid moiety onto CdiA-RBD^STECO31^ (*Alternate CDI Loci Arrangements*). Finally, Class V *E. coli* CdiA proteins (e.g., CdiA^SWW33^) recognize an as yet unidentified cell surface receptor. Interestingly, colicins are also known to utilize OMP receptors for target-cell entry (Cascales et al., 2007).

When CdiA is displayed on the inhibitor-cell surface, its RBD is presented at the most distal point to make contact with the target-cell while the FHA-1 domain and the YP-domain provide the scaffold necessary to extend CdiA away from the cell surface (Ruhe et al., 2018), Figure 1B. Though the N-terminal region of CdiA (N-terminus through the PT-motif) is highly conserved (77% identity for CdiA from EC93 and EC536), the RBD has decreased sequence identity (24% between EC93 and EC536) (Ruhe et al., 2017). Like the modularity observed for CdiA-CT, the RBD can be exchanged between CdiA proteins to alter target-cell receptor specificity (Ruhe et al., 2013; Ruhe et al., 2017). Notably, five distinct RBD sequences have been identified for *P. aeruginosa* (Allen and Hauser, 2019), however, no OMP receptor has been identified to-date.

As mentioned above, BamA is the target-cell receptor for Class I *E. coli* CdiA proteins. While BamA is highly conserved across enterobacteria with 73–93% sequence identity across *E. coli* EC93, *Salmonella* Typhimurium, *E. cloacae, Proteus mirabilis, Citrobacter freundii,* and *Enterobacter aerogenes,* the regions with highest sequence variability in enterobacteric BamA are in the extracellular loops (Ruhe et al., 2013). Strikingly, the exchange of *E. cloacae* extracellular loops six and seven for *E. coli* loop sequences allowed for chimeric *E. cloacae* BamA to be recognized by *E. coli* CdiA with subsequent CdiA-CT translocation (Ruhe et al., 2013). This suggests that BamA loops six and seven appear to be the only requirement for target-cell recognition for Class I *E. coli* CdiA effectors.

### Current Understanding of CDI Toxin Delivery

When CdiB presents CdiA onto the surface of the cell, the C-terminus of CdiA is retained in the inhibitor-cell periplasm until the CdiA-RBD binds its cognate target-cell receptor (Ruhe et al., 2017; Lin et al., 2020), Figure 1B. With target-cell receptor recognition, inhibitor-cell CdiA secretion continues, resulting in the translocation of CdiA-PT and CdiA-CT regions into the periplasm of the target-cell. The translocation event relies on contacts between FHA-2 and the target-cell; however, the precise mechanism of target-cell outer membrane translocation is unclear. As there is evidence that FHA-2 is internalized by the target-cell, and FHA-2 is predicted to structurally resemble a β-barrel OMP involved in lipopolysaccharide transport (*Shigella* LptD), it has been postulated that FHA-2 forms a β-barrel pore in the target-cell membrane for CdiA-CT toxin translocation (Ruhe et al., 2018).

Once the CdiA-PT and CdiA-CT regions enter the target-cell periplasm, cleavage of the CdiA-CT region from the large CdiA N-terminal region has not been well characterized. However, the PT-motif could signal for cleavage. Interestingly, in some *Pseudomonas* strains, a bacterial intein-like (BIL) domain was identified that terminates just before the CdiA-CT region (Mercy et al., 2016). As BIL domains have been implicated in autoproteolytic cleavage in *P. syringae* CdiA-CT (Amitai et al., 2003), similar domains could be present in other bacteria. Notably, the *Pseudomonas* WVHN PT-motif is predicted to be involved in BIL domain protein cleavage, where the terminal Asn residue is the C-terminal residue of the N-terminal domains following cleavage and a putative active site residue in the *P. syringae* CdiA BIL domain.

The translocation of the CdiA-CT region from the periplasm into the target-cell cytosol is dependent on the CdiA-CT entry domain, which recognizes a specific IMP and dictates the mechanism of cytosol entry (Willett et al., 2015a; Lin et al., 2020). Known CDI IMP receptors are multidrug transport protein component, AcrB (Aoki et al., 2008); ABC transporter membrane permeases MetI, RbsC, and GltJ/GltK; phosphotransferase, PtsG, and the ATP-dependent zinc metallopeptidase, FtsH; and an uncharacterized IMP, YciB (Willett et al., 2015a); and SecY, the channel-forming subunit for Sec pathway IMP internalization (Jones et al., 2021). Lastly, the IMP complex, DppB/DppC, part of an ABC dipeptide import system, was required for the cytosolic entry of a CdiA-CT in *P. aeruginosa* (Allen et al., 2020), however, as nine different translocation domains have been identified in *P. aeruginosa* (Allen and Hauser, 2019), other IMPs likely mediate CdiA-CT translocation. Additionally, the energy to cross the target-cell inner membrane requires proton-motive force (pmf) (Ruhe et al., 2014). Interestingly, the translocation of colicin toxins across the outer membrane is similarly dependent on a proton gradient (Cascales et al., 2007). However, CdiA does not require pmf for outer membrane translocation, further delineating the outer and inner membrane translocations steps (Ruhe et al., 2014).

Recently, the structure of a MetI-dependent CdiA-CT entry domain from *E. coli* STEC_MHI813 was characterized by NMR (Bartelli et al., 2019). The structure revealed a predominantly helical protein with a dynamic N-terminal region structured by disulfide bonds that are required for CdiA-CT translocation into the target-cell cytosol. Strikingly, these disulfide-forming cysteine residues are conserved across CdiA-CT entry domains while most CdiA proteins are otherwise devoid of cysteine residues. The prevalence of entry domain disulfide bonds suggests widespread importance for disulfide bonds in transport to the cytosol. Further, the CdiA-CT entry domain adopts a molten helical structure (Bartelli et al., 2019) akin to the increased flexibility of some colicin proteins in the presence of anionic lipids (Cascales et al., 2007). This physical property is also likely important in the transport of some CdiA-CT toxins across target-cell membranes aiding in translocation upon contact with membrane anionic lipids.

Substantial ground has been made in characterizing the mechanism of CdiA-CT delivery into the target-cell, however significant questions remain. In particular, the precise determinants of how CdiA-CT is translocated across the target-cell outer and inner membrane, and the molecular details behind the cleavage event that frees CdiA-CT from CdiA are still unknown. Much work is required to understand the mechanism of toxin cleavage and translocation.

### The Structure of the CDI Two-Partner Secretion Protein, CdiB

CdiA is presented onto the surface of CDI^+^ bacteria by the TpsB protein CdiB, which is an Omp85 family member. The structures of CdiB from *Acinetobacter baumannii* and *E. coli* (Guerin et al., 2020) have been solved and each comprise of a 16-stranded β-barrel with an N-terminal α-helix H1 that transverses the center of the β-barrel from extracellular to periplasm side followed by two N-terminal periplasmic POTRA domains and a canonical extracellular loop L6 (Figure 1C). Based on similarity to other TpsB proteins (Clantin et al., 2007; Gruss et al., 2013; Noinaj et al., 2013; Ni et al., 2014; Maier et al., 2015; Guerin et al., 2020), L6 is proposed to act as a ‘lid-lock’ inside the β-barrel and is probably essential for CdiA secretion to the inhibitor-cell surface. Further, POTRA domain P2 has been implicated in CdiA recognition and secretion (Hodak et al., 2006). Even though CdiA proteins are highly homologous, CdiA secretion appears to be species specific, wherein CdiA from *A. baumannii* cannot be secreted by *E. coli* CdiB, and similarly, *E. coli* CdiA is not displayed on the cell-surface in the presence of *A. baumannii* CdiB (Guerin et al., 2020). Notably, CdiB structures have high similarity to that of FhaC, an OMP responsible for the secretion of the FHA toxin (Clantin et al., 2007; Maier et al., 2015). Thus, research on FhaC is likely relevant to CdiB. The structures of CdiB show a “closed” conformation with α-helix H1 blocking the lumen of the β-barrel (Guerin et al., 2020). This internal helix may be important in ensuring unidirectional CdiA secretion. Questions remain as to how α-helix H1 is displaced to allow for CdiA secretion and what processes would be required to make this movement energetically favorable.

## Genetics of CDI and Contact-Dependent Signaling

The three genes, *cdiA, cdiB* and *cdiI*, responsible for CDI are encoded together in a *cdi* gene cluster (Figure 1A). The two best characterized CDI systems are those from *Burkholderia* and *E. coli*; strikingly, the *cdi* loci in these two organisms have different genetic organization. The simplest *cdi* loci in γ-proteobacteria like *E. coli* is organized as *cdiBAI* (Willett et al., 2015b; Beck et al., 2016), while in *Burkholderia* species and most β-proteobacteria (excluding *Neisseria*) it is organized as *cdiAIB* (or *bcpAIB*) (Anderson et al., 2012; Nikolakakis et al., 2012). Interestingly, while only ∼25% of *E. coli* species possess a *cdi* gene cluster, 100% of the sequenced isolates of *B. pseudomallei*, *P. aeruginosa, Neisseria meningitidis,* and *Yersinia pestis* strains have at least one *cdi* locus (Poole et al., 2011; Anderson et al., 2012; Nikolakakis et al., 2012; Willett et al., 2015b; Beck et al., 2016; Mercy et al., 2016; Allen and Hauser, 2019). Further, many species possess multiple *cdi* loci: *B. pseudomallei* strains have as many as three *cdi* gene clusters (Anderson et al., 2012; Nikolakakis et al., 2012), 81% of *P. aeruginosa* strains have two *cdi* loci (Mercy et al., 2016; Allen and Hauser, 2019), and *Acinetobacter baumannii DSM30011* contains two *cdi* loci (Roussin et al., 2019). CDI^+^ bacterial species have either *Burkholderia* or *E. coli cdi* loci organization, and this organization is consistent within bacterial strains of the same bacterial species.

### Alternate CDI Loci Arrangements

Many *cdi* loci are complicated by accessory genes, transposable elements, and the presence of orphan toxin and immunity protein modules (Poole et al., 2011). Indeed, in *Burkholderia* ten distinct *cdi* locus types have been described (Anderson et al., 2012; Nikolakakis et al., 2012). Most *B. pseudomallei* strains encode a *bcpO* gene in their *cdi* cluster, resulting in a *bcpAIOB* loci. The accessory protein BcpO localizes to the inner leaflet of the outer membrane, and *bcpO* mutants in *B. thailandensis* are associated with reduced CDI activity and a defect in autoaggregation (Anderson et al., 2012). BcpO is a predicted lipoprotein but has not been well characterized and its specific activity is unknown.

Class IV *E. coli* CdiA proteins are encoded by the *cdiBCAI* type loci, encoding a CdiC accessory protein (Willett et al., 2015a; Ogier et al., 2016). As CdiC bears sequence similarity to the hemolysin activator (HlyC), CdiC is a predicted lysyl acyltransferase that potentially activates CdiA through lysine acetylation (Ogier et al., 2016). Recently, it has been shown that *E. coli* STEC_O31 CdiC appends 3-hydroxydecanoate to a specific lysine residue within CdiA-RBD^STECO31^ (Halvorsen et al., 2021). As the loss of CdiA-RBD^STECO31^ acylation resulted in reduced toxicity to the target-cell, and Class IV CdiA-RBD recognize LPS on the target-cell, it seems likely that CdiA-RBD acylation could be involved in outer membrane entry, increasing the affinity and stabilizing the interaction of CdiA^STECO31^ with the target-cell LPS-rich leaflet (Halvorsen et al., 2021). The precise mechanisms of CdiA lipidation by CdiC and CdiA fatty acid insertion into the target-cell outer membrane remain unknown.

### Orphan Toxin and Immunity Genes at CDI Loci

Many organisms, like *E. coli, Y. pestis,* and *P. aeruginosa*, encode a series of orphan toxin (CdiA-CT) and immunity (CdiI) modules downstream of the *cdi* loci (Poole et al., 2011; Allen and Hauser, 2019). *Burkholderia* species sometimes encode these modules between *cdiI* and *cdiB* (Anderson et al., 2012; Nikolakakis et al., 2012). While the orphan immunity genes appear to be expressed, the orphan toxin genes lack a translation initiation codon, typically starting at the PT-motif (VENN (E/Q)LYN, or other) (Poole et al., 2011). These orphan toxin modules have toxic activity when expressed (Poole et al., 2011), thus, reorganization of the *cdi* loci could result in novel CdiA proteins encoding orphan toxin modules. These orphan toxin/immunity modules may represent ancestral toxin/immunity pairs that were displaced through the introduction of new toxin/immunity pairs (Jones et al., 2017). Some modules have homology to toxin and immunity proteins from other bacterial strains and are perhaps evidence of horizontal gene transfer (HGT). Furthermore, orphan immunity proteins likely provide protection against alternative CDI systems. Because immunity to additional CDI systems would confer evolutionary advantages, an organism would likely face selective pressures to maintain additional *cdiI* genes. Such evolutionary pressure would explain the observation that *cdi* loci in *P. aeruginosa* strains have as many as 15 orphan immunity genes (Allen and Hauser, 2019). Similarly, analysis of a *S.* Typhi genomic islet revealed an encoded orphan CdiI protein homologous to an *E. coli* O157:H7 strain EC869 CdiI (Barretto and Fowler, 2020), while the rest of the *S.* Typhi is devoid of *cdi* genes. This finding suggests that *S.* Typhi encodes the *cdiI* gene to protect itself against competing bacteria that encode EC869-like toxins.

### Contact-Dependent Signaling

Recently, mobile genetic elements were characterized in *B. thailandensis* that carry *bcpAIOB* on extrachromosomal DNA “megacircles” (Ocasio and Cotter, 2019). In these megacircles, the *cdi* locus is framed by IS2-like elements, allowing for insertion of CDI genes into chromosomes. While these megacircles have not been observed in other bacteria, this system shows a mechanism by which CDI genes can be replicated and inserted directly into DNA. Further these transposable megacircles have been implicated in contact-dependent signaling (CDS) (Ocasio and Cotter, 2019).

While CDI describes the competition and discrimination bacteria impose against “nonself” cells, CDS describes a phenomenon where CDI proteins mediate cooperative behaviors amongst “self’ or sibling cells (Danka et al., 2017; Ocasio and Cotter, 2019). CDS behaviors include the formation of DNA megacircles, along with biofilm formation, pigmentation, and polysaccharide production in *Burkholderia* species (Garcia et al., 2013; Garcia et al., 2016; Ocasio and Cotter, 2019). CDS is an emerging field of investigation, and other organisms need to be investigated for CDS phenotypes.

## Functional and Structural Diversity of CDI Toxins and Their Immunity Proteins

Currently, at least 130 CdiA-CT diverse sequence types have been identified (Jamet and Nassif, 2015), 42 of which have predicted biochemical activities and/or Pfam annotations. While multiple unique catalytic activities have been identified for CdiA-CT toxins *in silico* (Allen and Hauser, 2019; De Gregorio et al., 2019), only a small subset have been experimentally characterized. The first experimentally characterized CDI toxin was *E. coli* EC93 CdiA-CT, a pore-forming toxin that dissipates pmf in the target-cell membrane, leading to cell death (Aoki et al., 2005; Aoki et al., 2009). However, most CDI toxins are nucleases, where different CDI toxins show specificity for various nucleic acid substrates, cleavage-sites, and required cofactors (Poole et al., 2011; Morse et al., 2012; Beck et al., 2014; Morse et al., 2015; Johnson et al., 2016a; Johnson et al., 2016b; Batot et al., 2017; Jones et al., 2017; Michalska et al., 2017; Gucinski et al., 2019; Allen et al., 2020) (Table 1).

As discussed above, CdiA-CT is comprised of two domains: the N-terminal entry domain signals for IMP recognition and toxin translocation, and the most C-terminal domain possesses cytotoxic activity (Figure 1A). While a structure of a CdiA-CT entry domain was recently characterized by NMR (Bartelli et al., 2019), many CdiA-CT cytotoxic domains from various bacterial species have been structurally and biochemically characterized. Here, we will discuss our current structural and biochemical knowledge of the CdiA-CT cytotoxic domain (hereon referred to as CdiA-CT).

Currently, there are biochemically characterized toxins and solved X-ray crystal structures of toxin/immunity protein complexes from *Burkholderia pseudomallei* E479 (CdiA-CT/CdiI^E479^) (Johnson et al., 2016b), *B. pseudomallei* 1026b (CdiA-CT/CdiI^1026b^) (Morse et al., 2012)*, E. cloacae* ATCC 13047 (CdiA-CT/CdiI^ECL^) (Beck et al., 2014), *E. coli* 3006 (CdiA-CT/CdiI^EC3006^) (Gucinski et al., 2019), *E. coli* NC101 (CdiA-CT/CdiI^NC101^) (Jones et al., 2017), *E. coli* STEC_O31 (CdiA-CT/CdiI^STECO31^) (Michalska et al., 2018), *E. coli* TA271 (CdiA-CT/CdiI^TA271^) (Morse et al., 2012), uropathogenic *E. coli* 536 (CdiA-CT/CdiI^UPEC536^) (Johnson et al., 2016a), *K. pneumoniae* 342 (CdiA-CT/CdiI^Kp342^) (Gucinski et al., 2019), *P. aeruginosa* PABL017 (CdiA-CT/CdiI^PABL017^) (Allen et al., 2020), *Yersinia kristensenii* (CdiA-CT/CdiI^Ykris^) (Batot et al., 2017) and *Yersinia pseudotuberculosis* YPIII (CdiA-CT/CdiI^YpIII^) (Morse et al., 2015) (Table 1). Each structure has a unique toxin/immunity interface, where the immunity protein neutralizes the toxin by physically blocking substrate access through direct interaction with the active site, or an exosite through an unknown mechanism. As the majority of the CdiA-CT toxin structures appear to be members of the BECR (Barnase-EndoU-Colicin E5/D-RelE) or the PD-(D/E)XK superfamily, these toxins will be grouped and discussed by their superfamily designation.

### BECR Family CdiA-CT Toxins and Their Complexes

**FIGURE 2:**
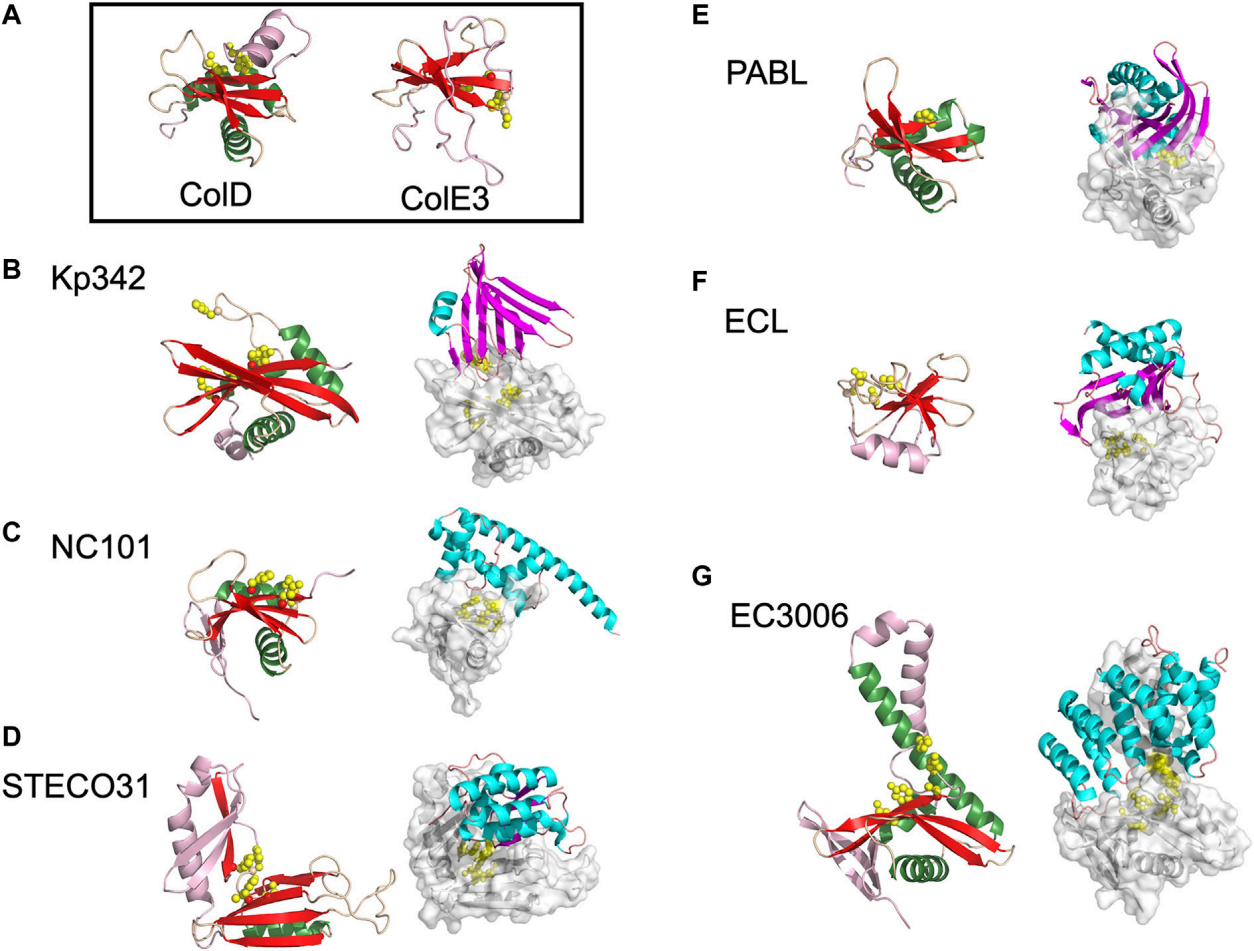
The diverse range of structures for BECR family CdiA-CT toxins and their CdiA-CT/CdiI complexes. **(A)** As representatives of BECR structural folds we show and box ColD (PDB ID: 5ZNM (Chang et al., 2018)) and ColE3 (PDB ID: 2B5U). ColD and ColE3 are colored as in the left panels described below. CDI BECR toxin and toxin/immunity complex structures are shown from: **(B)** *K. pneumoniae* 342 (Kp342) [PDB ID: 6CP9 (Gucinski et al., 2019)], **(C)** *E. coli* NC101 [PDB ID: 5I4Q (Jones et al., 2017)], **(D)** *E. coli* STEC_O31 [PDB ID: 5HKQ (Michalska et al., 2018)], **(E)** *P. aeruginosa* PABL017 [PDB ID: 6D7Y (Allen et al., 2020)], **(F)** *E. cloacae* ATCC 13047 (ECL) [PDB ID: 4NTQ (Beck et al., 2014)], and **(G)** *E. coli* 3006 (CdiA-CT/CdiI^EC3006^) [PDB ID: 6CP8 (Gucinski et al., 2019)]. For each pair, the left panel displays the toxin alone in cartoon representation with its BECR core structure colored by secondary structure with β-strands in red, α-helices in green, and loops in wheat, and the remainder of the secondary elements are colored in light pink. Active site residues are shown as yellow spheres. In the right panel is the CdiA-CT/CdiI complex with CdiI colored by secondary structure: β-strands in magenta, α-helices in cyan, and loops in salmon. CdiA-CT has a semi-transparent molecular surface with white cartoon representation and with active site residues shown as yellow spheres.

The BECR superfamily is incredibly diverse and includes barnase, a ribonuclease toxin from *Bacillus amyloliquefaciens;* the EndoU (Endoribonuclease specific for uridylate) superfamily of nucleases; colicins ColE5, ColD and ColE3 (Figure 2A), discussed earlier, with tRNase or rRNase activity; and the RelE/ParE superfamily, which includes the RelE family of ribosome dependent mRNA endonucleases and the ParE family of plasmid partition proteins that inhibit DNA gyrase and DNA replication. Characterized CdiA-CT toxin structures have homology to proteins from each of these BECR subfamilies apart from barnase (Table 1).

While several CDI toxins have sequence similarity to BECR nucleases, most structurally characterized BECR CdiA-CT proteins were only recognized as BECR family members based on their structure and not primary sequence alone. To date, CdiA-CT^EC3006^, CdiA-CT^Kp342^, CdiA-CT^NC101^, CdiA-CT^STECO31^, CdiA-CT^ECL^, CdiA-CT^PABL017^, and CdiA-CT^Ykris^ all have structural homology to BECR family members (Table 1; Figure 2). BECR-fold CdiA-CT proteins highlight the diversity of the BECR-fold family and the difficulty in identifying BECR family members and/or their specific nuclease activity by sequence alone.

Despite reasonable structural similarities, where CdiA-CT proteins typically align to BECR core domains with 2.4–3.5 Å root mean square deviation (rmsd), the sequence identity between these proteins is low with 7–17% sequence identity (Table 1). The BECR CdiA-CTs all have nuclease activity and comprise the BECR α/β-core, however, like the canonical BECR family members, the BECR α/β-core is marked by modifications, insertions and substitutions of secondary structure elements that are distinct for each CdiA-CT. This phenomenon has also been observed in non-CDI PTS toxins (Zhang et al., 2014). Interestingly, CdiA-CT^Kp342^ and CdiA-CT^NC101^ structurally resemble ColD, ColE5, and RelE toxin family members (BrnT, RelE, ParE) (Table 1; Figures 2A–C). Like CdiA-CT^Kp342^ and CdiA-CT^NC101^, other BECR CdiA-CTs have structural homology to several distinct proteins of the BECR superfamily. However, some toxins more closely resemble a single BECR family, where CdiA-CT^STECO31^ only has structural homology to EndoU type toxins, and CdiA-CT^ECL^ only resembles ColE3 (Figures 2D,F). Despite differences between BECR CdiA-CTs, structural similarity to canonical BECR toxins has guided the elucidation of substrate selectivity and the mechanism of action for each toxin.

#### Activities of BECR CdiA-CT Toxins

All the characterized BECR type CdiA-CT proteins have tRNase activity except for the rRNase CdiA-CT^ECL^. However, there are striking differences in substrate specificity, active site residues and cofactor requirements.

CdiA-CT^Kp342^ and CdiA-CT^EC3006^ are isoacceptor-specific tRNases with specificity for the anticodon loop of tRNA_GAU_^Ile^ (Willett et al., 2015a; Gucinski et al., 2019). While both toxins recognize the same acceptor stem, they have slightly shifted cleavage sites as CdiA-CT^EC3006^ cleaves after nucleotide C71, while CdiA-CT^Kp342^ cleaves after nucleotide C72. Notably, both toxins show specificity for deacylated or uncharged tRNA; this perhaps unprecedented substrate specificity may reflect cellular populations of tRNA in bacteria, as ∼50% of *E. coli* tRNA was observed to be deacylated (Gucinski et al., 2019). Both toxins have structural homology to ColD (Table 1), and in turn, active site residues were identified through homology to the well-characterized ColD toxin (Figures 2A,B,G) (Chang et al., 2018). Mutational analysis of CdiA-CT^Kp342^ and CdiA-CT^EC3006^ suggests that they employ a Lys-Tyr-Thr catalytic triad, in addition to a key histidine residue and arginine residue(s), required for tRNase activity (Table 1). A notable difference between the two toxins is that, while the activity of CdiA-CT^EC3006^ is independent of other proteins, CdiA-CT^Kp342^ activity was shown to require the translation elongation factors EF-Tu and EF-Ts proteins as well as the nucleotide guanosine-5′-triphosphate (GTP)—an observation we will revisit in *Toxin Activation by Target-Cell Proteins*.

Like CdiA-CT^Kp342^, CdiA-CT^NC101^ requires EF-Tu, EF-Ts and GTP for activity. However, CdiA-CT^NC101^ has an active site that includes His248 and Arg200 and cleaves several different tRNAs, where substrate specificity stems from a guanine discriminator nucleotide adjacent to the cleavage site (Jones et al., 2017). The activity of CdiA-CT^PABL017^ is relatively uncharacterized, but has a key catalytic histidine residue, His3372, and preferentially cleaves tRNA^Gln^ and tRNA^Pro^ *in vitro* (Allen et al., 2020), Figure 2E. Finally, CdiA-CT^STECO31^ has homology to EndoU proteins, but lacks the uridylate-specific tRNase activity characteristic of the EndoU family (Michalska et al., 2018). Like other the EndoU nucleases, the nuclease domain of CdiA-CT^STECO31^ has two α/β subdomains with a common catalytic His-His-Lys triad in the N-terminal subdomain. Evolutionary analysis and RNase experiments places CdiA-CT^STECO31^ in the clade III EndoU subfamily, acting as metal-independent anticodon loop endonuclease that preferentially cleaves tRNA_UUC_^Glu^.

The only structurally characterized BECR CdiA-CT protein without tRNase activity is CdiA-CT^ECL^. Strikingly, CdiA-CT^ECL^ only has homology to ColE3 (Table 1; Figures 2A,F). Like ColE3 (Lancaster et al., 2008; Ng et al., 2010), CdiA-CT^ECL^ is a potent rRNase, cleaving target-cell 16S rRNA between A1493 and G1494 (*E. coli* numbering for 16S rRNA) and resulting in protein translation inhibition (Beck et al., 2014). The active site of CdiA-CT^ECL^ is composed of Asp203, Asp205, His207 and Lys214, and aligns well to the active site of ColE3. Other CdiA-CT proteins are predicted to have similar function to ColE3 and CdiA-CT^ECL^. Indeed, the prediction that CdiA-CT from *Erwinia chrysanthemi* EC16 (CdiA-CT^EC16^) has 16S rRNase activity as it shares the same catalytic motif as ColE3 and CdiA-CT^ECL^ (Walker et al., 2004) was confirmed by biochemical characterization (Beck et al., 2014).

#### BECR CdiA-CT/CdiI Complexes

There is little in common between the BECR CdiA-CT immunity proteins. For example, CdiI^Kp342^ is almost entirely composed of β-strands and, CdiI^EC3006^ is completely α-helical (Gucinski et al., 2019) (Figures 2B,G). The toxin/immunity interfaces are also distinct with CdiA-CT buried surface area ranging from 775 Å^2^ to 1928 Å^2^ or comprising 13–28% of the total toxin surface area for CdiA-CT/CdiI^NC101^ and CdiA-CT/CdiI^ECL^ complexes, respectively (Table 1 (Beck et al., 2014; Gucinski et al., 2019)). However, the interactions at the interface are generally quite similar, with a handful of salt bridges, a dramatic network of hydrogen bonds and a large number of van der Waals contacts (Table 1). These vast and highly coordinated toxin/immunity interfaces result in high affinity and highly specific complexes. This specificity is underlined as closely related CdiI proteins have never been successfully exchanged to protect a cell from non-cognate toxins.

While CdiI proteins sometimes have homology to known protein families, thus far no catalytic activity has been associated with an immunity protein. Instead, CdiI proteins appear to have evolved with the sole role of protecting isogenic bacteria from its cognate toxin. Closely related CdiA-CT proteins—for example, CdiA-CT^EC3006^ and CdiA-CT^Kp342^ that have the same structural fold and substrate, and similar active sites (Gucinski et al., 2019)—can have such dramatically structurally dissimilar immunity proteins that each CdiI appears to have evolved from a unique evolutionary ancestor (Figures 2B,G). Further, structurally similar immunity proteins often have significant sequence disparity in key residues at the CdiA-CT/CdiI interface; this is the case for other immunity proteins in the CdiI^Kp342^ family and in the TA271 toxin/immunity protein family (Morse et al., 2015)—discussed further in *The PD-(D/E)XK Family CdiA-CT Toxins and Their Complexes*. These differences indicate that, even in cases with structural similarity, cognate toxin/immunity specificity is likely maintained, and highlights the strong evolutionary pressures placed on the development of toxin/immunity pairs (Gucinski et al., 2019).

Similarly, CdiI^STECO31^ (Figure 2D) has structural homology to *N. meningitidis* MC58 CdiI (CdiI^MC58^), where the two proteins have 2.6 Å rmsd over 111 Cα atoms (Tan et al., 2015; Michalska et al., 2018). While the toxins from these systems are quite similar with 39% sequence identity and possess equivalent tRNase activity, CdiI^STECO31^ has very low sequence identity (∼16%) with CdiI^MC58^. Despite similarities between the toxins and structural similarity between the immunity proteins, exchanging CdiI^MC58^ for CdiI^STECO31^ does not protect cells from CdiA-CT^STECO31^ cytotoxic activity (Michalska et al., 2018), highlighting the high specificity immunity proteins have for their cognate toxin.

The immunity proteins of the BECR CdiA-CTs generally inactivate their cognate toxin by binding directly to the active site. Notably, each of the known BECR CdiA-CT/CdiI complex interfaces involves direct contact between a key CdiA-CT catalytic residue and the immunity protein. For example, all three residues associated with toxicity in CdiA-CT^NC101^ interact directly with CdiI^NC101^, and the CdiA-CT^ECL^ catalytic residue Asp205 interacts directly with CdiI^ECL^ (Table 1). By interacting directly with toxin active site residues, CdiI effectively occludes substrate access to the toxin resulting in toxin neutralization.

### The PD-(D/E)XK Family CdiA-CT Toxins and Their Complexes

Currently there are four CdiA-CT toxins with structural homology to the PD-(D/E)XK superfamily nucleases: CdiA-CT^Bp1026b^, CdiA-CT^E479^, and CdiA-CT^TA271^ and its closely related family member CdiA-CT^YPIII^ (Morse et al., 2012; Morse et al., 2015; Johnson et al., 2016b). The PD-(D/E)XK nuclease superfamily are a diverse set of proteins with extreme sequence variability (Knizewski et al., 2007; Steczkiewicz et al., 2012). For this reason, identifying PD-(D/E)XK family members by sequence alone is difficult. PD-(D/E)XK proteins are characterized by a αβββαβ core, well-conserved catalytic lysine and aspartate residues, and one or more metal-binding sites coordinated by carboxylate groups (i.e., Asp or Glu residues). The proteins have diverse nuclease related functions, including amongst others: DNA restriction, tRNA splicing, transposon excision, DNA recombination and Holliday junction resolution (Knizewski et al., 2007; Steczkiewicz et al., 2012).

**FIGURE 3:**
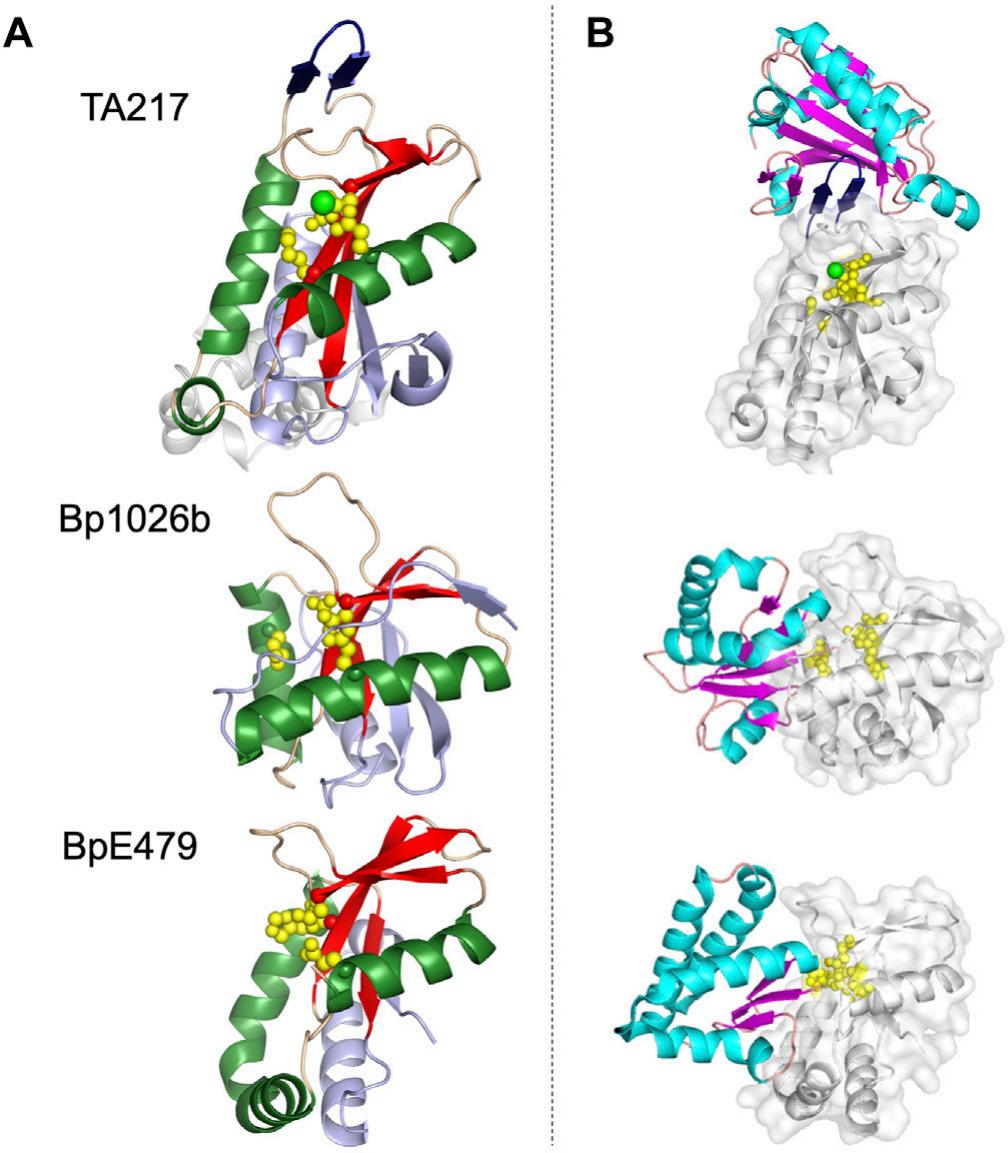
Representative structures of the PD-(D/E)XK family CdiA-CT toxins and CdiA-CT/CdiI complexes from *E. coli* TA271 (CdiA-CT/CdiI^TA271^) [PDB ID: 4G6U (Morse et al., 2012)], *B. pseudomallei* 1026b (CdiA-CT/CdiI^1026b^) [PDB ID: 4G6V (Morse et al., 2012)], and *B. pseudomallei* E479 (CdiA-CT/CdiI^E479^) [PDB ID: 5J4A (Johnson et al., 2016b)]. **(A)** CdiA-CT in cartoon representation with the core PD-(D/E)XK structure colored by secondary structure with β-strands in red, α-helices in green, and loops in wheat, and the remainder of the secondary elements colored in light blue. Notably, the structure of TA271 includes additional structure at the N-terminus (gray) domain, a Zn^2+^ ion (green sphere), and the TA271 core is interrupted by a protruding β-hairpin (dark blue). **(B)** Structures of PD-(D/E)XK family CdiA-CT/CdiI complexes. CdiI is colored by secondary structure: β-strands in magenta, α-helices in cyan, and loops in salmon. CdiA-CT is in white cartoon representation with a semi-transparent molecular surface and active site residues shown as yellow spheres. In the TA271 complex the bound metal ion and the TA271 β-hairpin are colored as in the toxin alone. The extra N-terminal region of TA271 is omitted.

Structural similarities between PD-(D/E)XK CdiA-CT toxins is readily apparent (Figure 3). CdiA-CT^TA271^ and CdiA-CT^YPIII^ belong to the same toxin/immunity family with a rmsd of 1.7 Å and high sequence identity (64%); the *Burkholderia* CdiA-CT^Bp1026b^ and CdiA-CT^E479^ are also similar with 2.9 Å rmsd and medium sequence similarity (19%) (Table 1). While these two pairs are disparate in sequence (5–12% identity), the two groups bear striking structural homology to each other with 3.5–4.4 Å rmsd. All four proteins have the conserved αβββαβ core (Table 1; Figure 3). The crystal structure of CdiA-CT^TA271^ is unique as preceding the C-terminal nuclease domain is an N-terminal helical domain, which is likely a portion of the N-terminal cytoplasmic entry domain (CdiA-CT entry domain) (Figure 3A). PD-(D/E)XK family members frequently have core structure variability in β-strand length, angling of the α-helices, and insertions of additional secondary structure elements into the core (Knizewski et al., 2007; Steczkiewicz et al., 2012). Though the α-helices in all the characterized PD-(D/E)XK CdiA-CTs are angled similarly in relation to the central β-sheet, the final two β-strands for CdiA-CT^TA271^ (and CdiA-CT^YPIII^) are extended when compared to those in the *Burkholderia* toxin structures (Figure 3). Other differences include an extended, dramatically curved C-terminal core α-helix in CdiA-CT^E479^, and an insertion in CdiA-CT^TA271^ (and CdiA-CT^YPIII^) where the final α-helix is preceded by a short α-helix that falls along the bent CdiA-CT^E479^ α-helix trajectory. Despite reasonable structural homology between all the PD-(D/E)XK CdiA-CTs (Table 1), the CdiI^E479^ and CdiI^1026b^ immunity proteins share no significant sequence or structural homology with each other or with CdiI^TA271^ (and CdiI^YPIII^). CdiI^TA271^ and CdiI^YPIII^, which again belong to the same toxin/immunity family, share ∼50% sequence identity and bear significant structural homology (1.0 Å rmsd) (Morse et al., 2015).

The active site for closely related CdiA-CT^TA271^ and CdiA-CT^YPIII^ is fully conserved, containing Glu177, Asp198, Ser209 and Lys211 (TA271 numbering) (Morse et al., 2012; Morse et al., 2015). CdiA-CT^Bp1026b^ has a similar active site whereby Ser209 is an aspartate residue (Asp223) (Morse et al., 2012). The active site of CdiA-CT^E479^ is unusual in that the catalytic lysine residue has been replaced with a histidine residue (His275) (Table 1) (Johnson et al., 2016b). Despite similarity between the PD-(D/E)XK CdiA-CT active sites, these toxins show two distinct catalytic activities. CdiA-CT^TA271^ is a Zn^2+^-dependent DNase, completely degrading plasmid DNA in the presence of Zn^2+^ and shows very reduced DNase activity if the divalent metal is Mg^2+^ (Morse et al., 2012). CdiA-CT^YPIII^ is also a Zn^2+^-dependent DNase and is not activated by Mg^2+^ (Morse et al., 2015). In contrast, both CdiA-CT^Bp1026b^ and CdiA-CT^E479^ have tRNase activity. CdiA-CT^Bp1026b^ specifically cleaves tRNA^Ala^ at the aminoacyl-acceptor stem (Nikolakakis et al., 2012), resulting in an accumulation of uncharged tRNA and disrupting translation (Morse et al., 2012). Conversely, CdiA-CT^E479^ cleaves tRNA_2_^Arg^ at the T-loop between a conserved thymidine (T55) and pseudouridine (ψ56) (Nikolakakis et al., 2012). CdiA-CT^1026b^ and CdiA-CT^E479^ active sites overlay extremely well. Examining the size and shape of each active site revealed that CdiA-CT^E479^ has a wider active site pocket compared to CdiA-CT^1026b^ (12.6 Å vs. 10.4 Å, respectively), which may play a significant role in substrate specificity (Johnson et al., 2016b).

The toxin/immunity protein interface for all PD-(D/E)XK CdiA-CT/CdiI complexes are extensive. The four toxin/immunity complexes have interfaces with ∼960–1,170 Å^2^ (10–15%) toxin buried surface area and are marked by multiple salt bridges, 14–19 hydrogen bonds and hundreds of van der Waals interactions (Table 1). Notably, CdiA-CT^TA271^ (and CdiA-CT^YpIII^) has an additional β-hairpin inserted into the nuclease core (Figure 3A). For CdiA-CT/CdiI^TA271^ (and CdiA-CT/CdiI^YpIII^), the toxin is inactivated through a highly unusual mechanism of β-augmentation, wherein the toxin inserts this β-hairpin into the pocket of cognate immunity, producing a highly stable six-stranded antiparallel β-sheet where the toxin β-hairpin is sandwiched between two immunity β-hairpins (Morse et al., 2012; Morse et al., 2015). The formation of this β-sheet between the two proteins yields a highly specific and high affinity complex (Morse et al., 2012). Despite significant sequence identity and structural homology, CdiI^TA271^ and CdiI^YpIII^ do not confer protection to the non-cognate toxin (Morse et al., 2015). Notably, both CdiA-CT^1026b^ and CdiA-CT^E479^ lack this extended β-hairpin element and have a larger toxin/immunity interface (14–15% of the buried toxin surface area) than CdiA-CT/CdiI^TA271^ and CdiA-CT/CdiI^YpIII^ (10–12% buried toxin surface area) (Table 1). For Bp1026b and BpE479, immunity protein specificity appears to stem from unique distributions of electrostatic charges at the protein-protein interfaces, preventing the interaction between non-cognate toxin/immunity pairs.

The immunity protein for CdiA-CT^TA271^ (and CdiA-CT^YpIII^) binds at an alternate location to the immunity proteins of CdiA-CT^1026b^ and CdiA-CT^E479^ (Figure 3B). While CdiI^1026b^ and CdiI^E479^ directly coordinate toxin active site residues, effectively rendering the protein inactive, no similar interaction is observed in the TA271 and YpIII toxin/immunity complexes. Instead, for this family of toxin/immunity proteins the toxin active site residues remain solvent exposed and perhaps available to bind substrate. It is currently unknown how CdiI^TA271^ neutralizes its toxin. However, perhaps formation of the toxin/immunity complex could prevent or restrict toxin mobility upon binding DNA substrate, inactivating the toxin.

While all of the PD-(D/E)XK CdiA-CT/CdiI complex structures reveal heterodimeric oligomerization, experiments with CdiA-CT^E479^ indicate the toxin adopts higher order oligomerization in the presence of tRNA. Size exclusion chromatography with CdiA-CT^1026b^ and CdiA-CT^E479^ indicate 1:1 binding of CdiA-CT to tRNA, but while CdiA-CT^1026b^ forms a monomeric protein:tRNA complex, CdiA-CT^E479^ forms tetrameric protein:tRNA complex (Johnson et al., 2016b). These results were supported experimentally by fitting tRNA-docked CdiA-CT^1026b^ and CdiA-CT^E479^ models into small-angle X-ray scattering (SAXS) derived electron density envelopes of CdiA-CT^1026b^ and CdiA-CT^E479^ in complex with tRNA substrate (Johnson et al., 2016b). Thus, while CdiA-CT^1026b^ interacts with a single tRNA molecule as a monomer, CdiA-CT^E479^ tetramerizes to form a complex with four tRNA molecules (Johnson et al., 2016b).

As PD-(D/E)XK proteins are characterized by highly variable sequences and active-site plasticity, CDI systems are able to generate a diverse array of cytotoxic proteins with unique substrate specificity, nuclease activity and oligomerization. These characterized PD-(D/E)XK CdiA-CT toxins are likely representative of a number of uncharacterized CdiA-CT proteins that belong to this superfamily, however, alternative toxin activities and structures would not be unexpected.

### CdiA-CT^Ykris^, a Bacterial RNase a Family Member

The structure of the toxin/immunity complex from *Yersinia kristensenii* ATCC 33638 revealed that CdiA-CT is a member of the RNase A superfamily, which previously had only been observed in eukaryotes (Batot et al., 2017; Cuthbert et al., 2017). Though CdiA-CT^Ykris^ shares little sequence similarity with RNase A family members, CdiA-CT^Ykris^ adopts a kidney shape that is formed by two curved β-sheet domains, and strongly resembles several RNase A family members (Holloway et al., 2011; Thiyagarajan and Acharya, 2013; Lomax et al., 2014), Figure 4A. Like RNase A proteins, CdiA-CT^Ykris^ has metal-independent RNase activity. However, while RNase A proteins have a conserved His-Lys-His triad, the active site of CdiA-CT^Ykris^ comprises His175, Thr276, Tyr278, and Arg186, suggesting an alternate mechanism of ribonuclease action (Table 1). In CdiA-CT^Ykris^, these residues are required for full RNase activity and cyclic cytidine monophosphate (cCMP) hydrolysis, another RNase A family activity (Raines, 1998). However, the structural homology of CdiA-CT^Ykri*s*^ to the RNase A family, and its RNase and cCMP hydrolytic activities, support its classification as a novel bacterial member of the RNase A superfamily.

**FIGURE 4:**
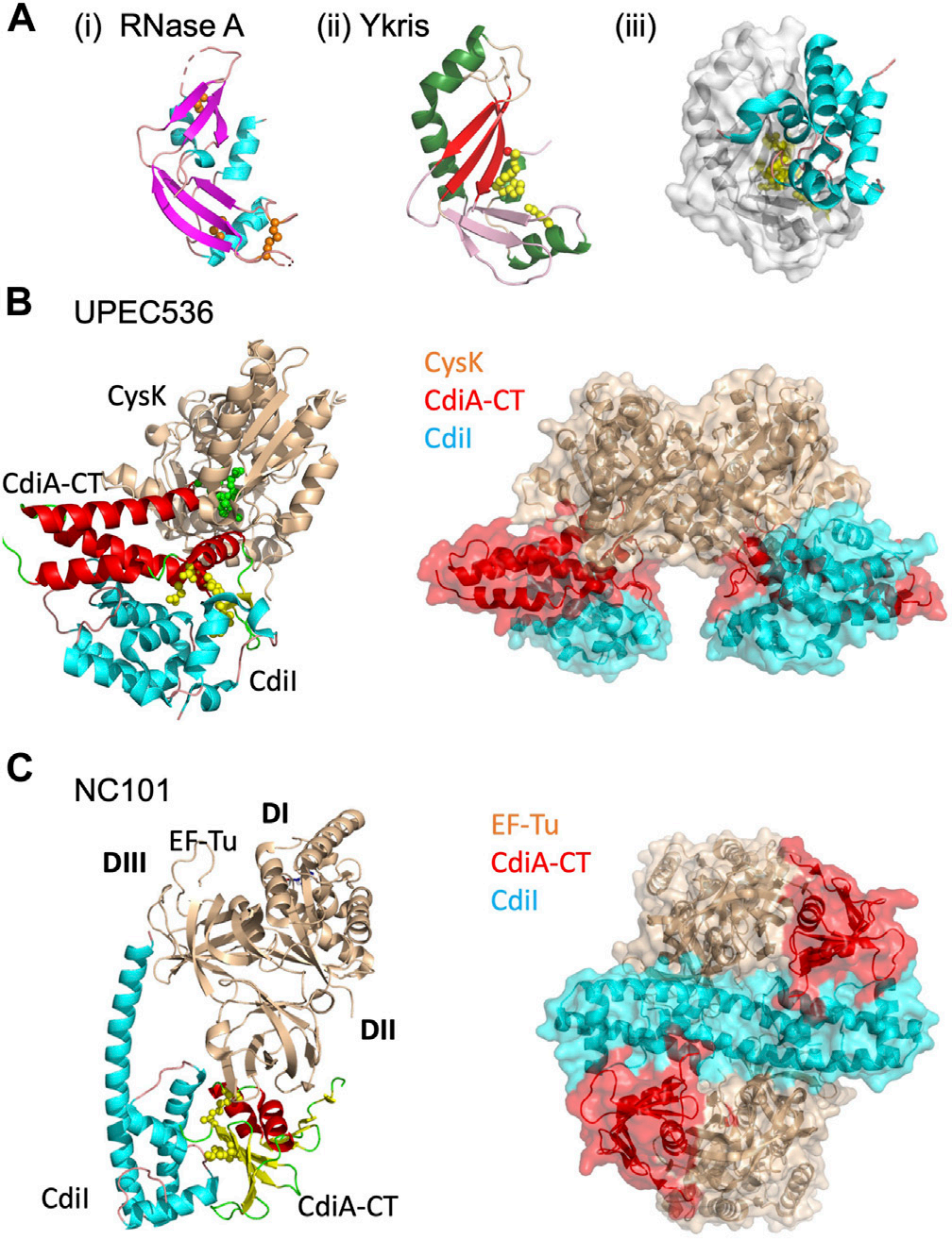
CdiA-CT toxins with unique characteristics. **(A)** *Y. kristensenii* (Ykris) [PDB ID: 5E3E (Batot et al., 2017)] has some structural homology to BECR family members, but most closely resembles RNase A [PDB ID: 4B36 (Thiyagarajan and Acharya, 2013)]. i) RNase A is shown in cartoon depiction with β-strands in magenta, α-helices in cyan, and loops in salmon, and with conserved disulfide bonds shown as orange spheres. ii) CdiA-CT^Ykris^ in cartoon representation with its BECR core structure colored by as in Figure 2 left panels. iii) The CdiA-CT/CdiI^Ykris^ complex with CdiI colored as in Figure 2 right panels. In **(B,C)** the accessory protein (CysK or EF-Tu) is shown in beige, while on the left the toxin and immunity protein are colored by secondary structure where CdiA-CT has β-strands in yellow, α-helices in red, and loops in green; CdiI has α-helices in cyan and loops in pink. Active site residues for CdiA-CT are shown as yellow spheres. In the complex on the right, CdiA-CT is colored red, CdiI cyan and the accessory protein is in beige. **(B)** In UPEC 536, CdiA-CT forms a complex with CdiI and CysK [PDB ID: 5J5V (Johnson et al., 2016a)]. The C-terminal residue of CdiA-CT (green spheres) that inserts into the CysK active site is highlighted. Shown on the right is the overall oligomerization of the CdiA-CT/CdiI/CysK complex, where CysK dimerization results in a dimer of heterotrimers. **(C)** In *E. coli* NC101, CdiA-CT interacts directly with CdiI and domain 2 (DII) of EF-Tu, while CdiI interacts with DII and domain 3 (DIII) of EF-Tu (PDB ID: 5I4R; (Jones et al., 2017). On the right, the CdiA-CT/CdiI/EF-Tu complex is shown, where CdiI forms the center of the dimer of heterotrimers.

Unlike eukaryotic RNase A proteins, CdiA-CT^Ykris^ has no disulfide bonds (Batot et al., 2017). Strikingly, RNase A undergoes cooperative unfolding at ∼60°C while CdiA-CT^Ykris^ gradually unfolds between 40 and 80°C (Batot et al., 2017). Thus, its thermal stability and lack of disulfide bonds enables CdiA-CT^Ykris^ to be more tolerant to unfolding/refolding than eukaryotic RNase A proteins. As CdiA-CT is transported from the periplasm of the CDI^+^ bacteria and through the target-cell outer and inner membranes to reach the target-cell cytosol, these biophysical characteristics of CdiA-CT^Ykris^ may allow for successful unfolding and refolding of the toxin in its passage through these environments.

Like other CdiA-CT/CdiI complexes, CdiI^Ykris^ forms an extensive interface with CdiA-CT^Ykris^ (Table 1; Figure 4Aiii). Like BECR-type CdiA-CT/CdiI complexes, CdiI^Ykris^ inactivates its cognate toxin through direct interaction with active-site residues: His175, Thr276, and Tyr278. Eukaryotes express an RNase A inhibitor (RI) to prevent RNase cytotoxicity (Dickson et al., 2005). Notably, CdiI^Ykris^ has no sequence or structural homology to RI.

Until the characterization of CdiA-CT^Ykris^ (Batot et al., 2017), the only known RNase A family members were in vertebrates (Dyer and Rosenberg, 2006). Extending the RNase A superfamily to include CdiA-CT^Ykris^, creates an RNase A subfamily with bacterial proteins/domains from both Gram-negative and Gram-positive bacteria, many of which play a role in bacterial virulence or competition and all of which have predicted immunity proteins and associated secretion systems (Batot et al., 2017).

## Toxin Activation by Target-Cell Proteins

### Discovery of Toxin Activation by Endogenous Target-Cell Proteins

While most CDI toxins possess active cytotoxic domains in the absence of a protein partner, some CDI toxins require one or more target-cell protein partners to activate the toxin. This phenomenon was first identified in the CDI system of uropathogenic *E. coli* 536 (UPEC536), which requires a target-cell metabolic protein to bind and activate its toxin. More recently, the discovery of several CDI systems that require both EF-Tu and EF-Ts for toxin activity suggests that toxin activation by endogenous target-cell proteins may be utilized by other CDI systems.

The requirement of a target-cell protein to activate delivered toxins might be more widespread than our current knowledge implies, but under sampled due to the difficulty of toxin protein partner detection and verification by *in vitro* methods. Further, interactions with endogenous cytosolic proteins may be another avenue through which CDI proteins have a significant role in contact-dependent signaling (CDS) (Danka et al., 2017; Ocasio and Cotter, 2019), where interactions between CdiA-CT/CdiI and specific cytosolic proteins in CDI^+^ cells could result in altered cell processes or gene regulation (Garcia, 2018).

### UPEC CDI Requires CysK for Toxin Activity

The CdiA-CT^UPEC536^ toxin is a potent tRNase that is activated upon binding to target-cell cysteine synthase A (CysK, *O*-acetylserine sulfhydrylase A), an enzyme involved in cysteine biosynthesis (Diner et al., 2012). CysK is extremely well conserved in Gram-negative bacteria, with ∼99% sequence identity between bacterial species (Burkhard et al., 1998; Rabeh and Cook, 2004; Claus et al., 2005). However, CysK is a non-essential enzyme as CysK-expressing cells also express an isoenzyme, CysM (Claus et al., 2005). In a target cell expressing cytosolic CysK, CdiA-CT^UPEC536^ binds to CysK with high affinity to form an active tRNase complex. Interestingly, CysM does not bind to CdiA-CT^UPEC536^, granting CysK-deficient cells immunity to CdiA-CT^UPEC536^. The CysK/CdiA-CT^UPEC536^ complex is inactivated upon forming a ternary complex with the cognate UPEC immunity protein (CysK/CdiA-CT^UPEC536^/CdiI^UPEC536^).

The structure of the CysK/CdiA-CT^UPEC536^/CdiI^UPEC536^ complex reveals an interesting mechanism of interaction between CysK and CdiA-CT^UPEC536^. The UPEC toxin interacts with CysK by inserting its C-terminal tail into the CysK active site cleft (Figure 4B) (Johnson et al., 2016a). This interaction mimics the native interaction between CysK and a serine acetyltransferase (CysE), another cysteine biosynthetic enzyme. CdiA-CT^UPEC536^ uses a conserved C-terminal Gly-X-Gly-Ile CysE motif to make similar contacts with CysK to those observed in the CysK/CysE complex (Francois et al., 2006; Zhao et al., 2006; Salsi et al., 2010; Diner et al., 2012; Wang and Leyh, 2012). The CysK-CdiA-CT^UPEC536^ complex cleaves cytosolic tRNA at the anticodon loop, rendering it unusable and halting protein translation in the cell (Diner et al., 2012). Notably, CysK is a homodimer and thus the ternary complex with CdiA-CT/CdiI^UPEC536^ forms a dimer of heterotrimers (Figure 4B).

The CdiA-CT^UPEC536^ structure is completely α-helical with no structural homology to any known tRNase to-date. Mutational and biochemical analysis revealed an active site consisting of Asp155, Trp176, His178, and Glu181 (Figure 4B) (Johnson et al., 2016a). Notably, Trp176 is involved in interactions with CdiI^UPEC536^, forming hydrophobic interactions with a hydrophobic patch on the immunity protein surface. The rest of the toxin/immunity interface is formed by a series of hydrogen bonds, including direct coordination of the toxin active site residues, His178 and Glu181.

To elucidate the mechanism of toxin activation by CysK, an Ntox28 homolog of CdiA-CT^UPEC536^ was identified from the Gram-positive *Ruminococcus lactaris*, Tox28^Rlac^. Tox28^Rlac^ is functionally homologous to CdiA-CT^UPEC536^ but does not require CysK for tRNase activity. Extensive experimental evidence shows that Tox28^Rlac^ is significantly more thermostable than CdiA-CT^UPEC536^ (Johnson et al., 2016a). Notably, upon CysK binding CdiA-CT^UPEC536^ becomes significantly more stable with an improved ability to bind tRNA substrate. This data suggests that CysK may act as a chaperone to CdiA-CT^UPEC536^. CdiA-CT toxins may be intrinsically unstable to allow the passage of partially unfolded toxin across bacterial membranes into the target-cell cytosol. Thus, cytosolic chaperones like CysK may be a necessary stabilizing scaffold for some CdiA-CT proteins to ensure a fully active toxin. The lack of disulfide bonds and increased thermal stability of the bacterial RNase A CdiA-CT^Ykris^ (*CdiA-CT* ^*Ykris*^ *, a Bacterial RNase a Family Member*) also supports this stability/folding hypothesis.

### EF-Tu and EF-Ts Are Necessary for Some CDI Systems

Following the discovery of the critical role of CysK in CdiA-CT^EC536^ activity, there was speculation on whether other CDI systems employ target-cell proteins in CdiA-CT activity. Recently, it was observed that the translation elongation factors EF-Ts and EF-Tu are involved in the tRNase activity of several *E. coli* CDI toxins: CdiA-CT^EC869^, CdiC-CT^96.154^, CdiA-CT^NC101^, and CdiA-CT^Kp342^ (Jones et al., 2017; Michalska et al., 2017; Gucinski et al., 2019). CdiA-CT^NC101^ and CdiA-CT^Kp342^ are both BECR CdiA-CT proteins and are discussed above in *BECR Family CdiA-CT Toxins and Their Complexes*. EF-Tu delivers aminoacyl-tRNAs (aa-tRNAs) to the free site of the ribosome during protein synthesis, while EF-Ts acts as a guanine nucleotide exchange factor for EF-Tu, catalyzing the release of guanosine-5′-diphosphate (GDP) from EF-Tu. CDI toxins that rely on these elongation factors are GTP-dependent tRNases and perhaps only recognize tRNA in an EF-Tu bound context or rely on EF-Tu to position tRNA correctly for toxin cleavage.

The CdiA-CT toxin of enterohemorrhagic *E. coli* 869 (CdiA-CT^EC869^) was the first CDI protein identified that requires EF-Ts and EF-Tu for tRNase activity *in vivo* (Jones et al., 2017). CdiA-CT^EC869^ requires EF-Tu and GTP to specifically cleave the 3′-end of the acceptor stem of tRNA^Gln^ and tRNA^Asn^, while EF-Ts further stimulates the tRNase activity of this complex (Jones et al., 2017). EF-Tu and EF-Ts are both necessary for CdiA-CT^NC101^ activity, which cleaves the single-stranded 3′-tail of the tRNA acceptor stem. Interestingly, CdiA-CT^NC101^ cleaves several tRNAs, where substrate specificity stems from a guanine discriminator nucleotide adjacent to the cleavage site. CdiA-CT^Kp342^, as discussed earlier, cleaves uncharged tRNA_GAU_^Ile^; this tRNase activity is greatly stimulated in the presence of both EF-Tu and EF-Ts (Gucinski et al., 2019). Notably, unlike CdiA-CT^EC869^ and CdiA-CT^NC101^, neither EF-Ts nor EF-Tu are required for CdiA-CT^Kp342^ activity as the same reaction can be carried out in their absence, albeit at a considerably slower rate.

While other BECR fold CdiA-CT toxin/immunity complexes are heterodimeric, both CdiA-CT/CdiI^Kp342^ and CdiA-CT/CdiI^NC101^ possess a homodimeric CdiI where each subunit binds one toxin, resulting in the formation of a heterotetramer or heterohexamer, depending on the inclusion of EF-Tu in the complex (Figure 4C). CdiI^Kp342^ is almost entirely composed of β-strands (Gucinski et al., 2019), and CdiI^NC101^ is completely helical (Jones et al., 2017) (Figures 2B,C); thus, CdiI homodimerization is quite distinct between these complexes. The CdiA-CT/CdiI^NC101^/EF-Tu complex is a dimer of heterotrimers, where CdiI forms a central dimer that interacts directly with EF-Tu and CdiA-CT (Jones et al., 2017), Figure 4C. The interaction between CdiI^NC101^ and its cognate toxin is much like other BECR CdiA-CT/CdiI complexes described above (Table 1; *BECR Family CdiA-CT Toxins and Their Complexes*). EF-Tu interacts with the N-terminal face of CdiA-CT^NC101^ through an extensive interface involving the formation of an anti-parallel 3-stranded β-sheet between the toxin and an EF-Tu β-hairpin alongside a vast network of interactions. Strikingly, a CdiA-CT^NC101^ Tyr192Arg mutation prevents the inclusion of EF-Tu into a complex with CdiA-CT/CdiI, and results in an inactive toxin. Docking CdiA-CT^Kp342^ onto EF-Tu at the CdiA-CT^NC101^/EF-Tu interface results in extensive clashes between CdiA-CT^Kp342^ and EF-Tu structural elements, suggesting that interactions between CdiA-CT^Kp342^ and EF-Tu are unique compared to the CdiA-CT^NC101^/EF-Tu complex (Gucinski et al., 2019).

Despite the crystal structure and biochemical analysis, there are still unanswered questions about how CdiA-CT^NC101^ cleaves tRNA in complex with EF-Tu. For example, when the CdiA-CT^NC101^/EF-Tu complex is superimposed onto a structure of the 5′-guanylyl imidodiphosphate (GDPNP, a non-hydrolysable GTP analog)/EF-Tu/aa-tRNA complex, the toxin active site is located more than 10 Å away from the tRNA cleavage site. Furthermore, the complexes do not superimpose perfectly as the superimposed toxin has intermolecular clashes with domain I of EF-Tu and bound aa-tRNA from the GDPNP/EF-Tu/aa-tRNA complex. Thus, CdiA-CT^NC101^ binding must impose structural changes to the GTP/EF-Tu/tRNA complex, likely resulting in the correct tRNA placement and exposure for cleavage by CdiA-CT^NC101^.

Both CdiA-CT/CdiI ^EC869^ and CdiA-CT/CdiI^NC101^ complexes copurify with EF-Tu to form high-affinity ternary complexes (Jones et al., 2017; Michalska et al., 2017). Notably, while CdiA-CT^96.154^ copurifies with EF-Tu, the complex is extremely unstable, indicating interactions between EF-Tu and CdiA-CT^96.154^ are likely weaker than those between EF-Tu and CdiA-CT^EC869^ or CdiA-CT^NC101^. The differences in affinity between CdiA-CTs and EF-Tu highlight the difficulty in identifying novel CdiA-CT effectors as many heretofore undetected interactions will not be observed under typical experimental conditions.

## Harnessing CDI to Fight Human Disease and Other Applications

Toxin-antitoxin (TA) systems are a prokaryotic intracellular stress response mechanism. The type II TA systems are the best characterized and consist of an antitoxin protein that binds and inactivates a toxin protein, which is akin to CDI toxin and immunity proteins. The application of type II TA systems for molecular biology, industrial and therapeutic applications have been explored and previously reviewed (Williams and Hergenrother, 2012; Unterholzner et al., 2013; Chan et al., 2015; Yeo et al., 2016). Successful strategies utilizing TA systems for human health and biotechnology represent an excellent framework for how CDI toxin and immunity protein could be harnessed to benefit human health.

**FIGURE 5:**
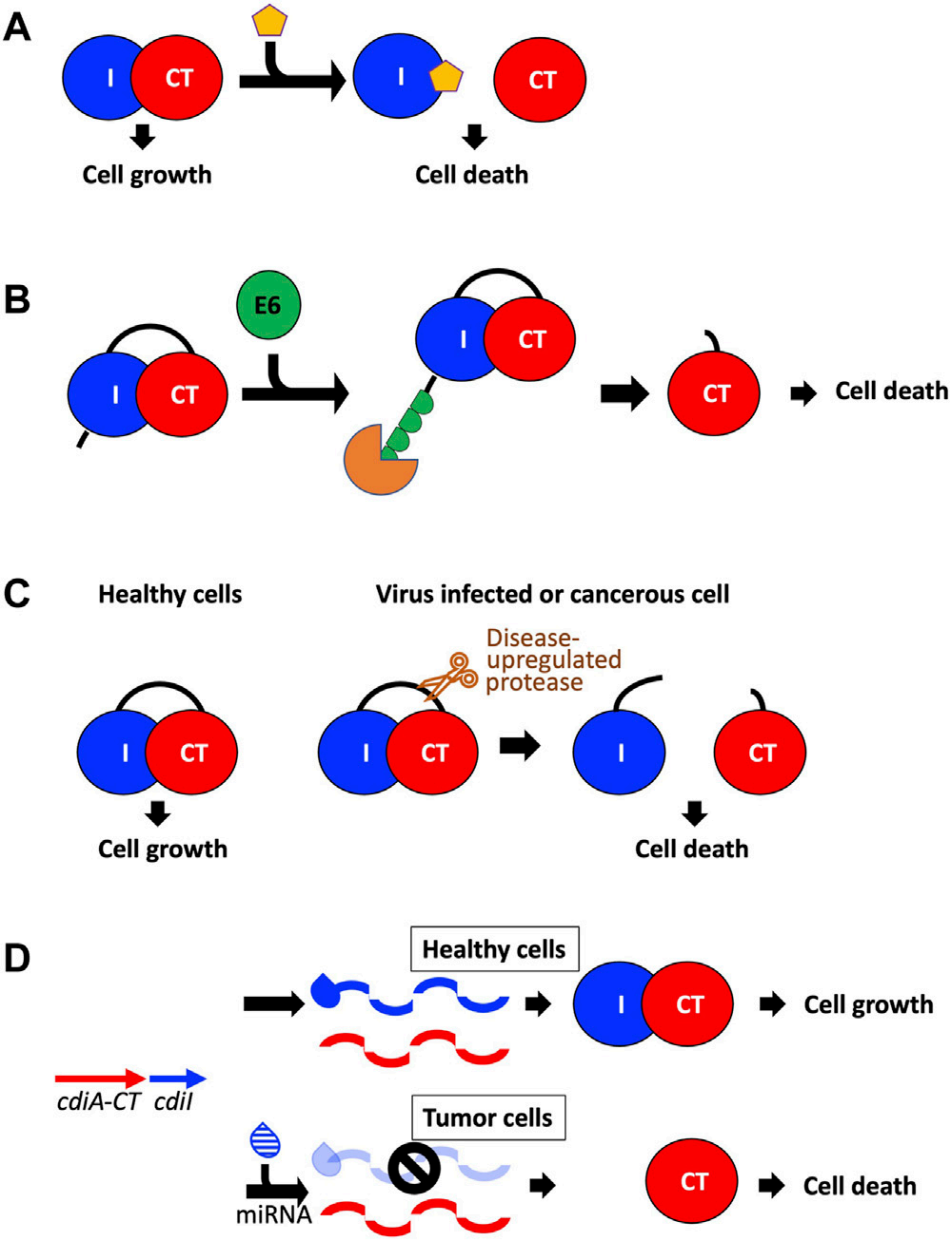
Potential avenues toward utilizing CDI toxins to treat human disease. **(A)** The toxin/immunity complex can be disrupted by designing small molecules that bind the immunity protein and free the toxin to initiate cell death (Williams and Hergenrother, 2012; Unterholzner et al., 2013; Morse et al., 2015). **(B)** A fusion toxin/immunity protein is generated that encodes an onco-specific POM (osPOM) at the immunity N-terminus that signals for oncoprotein E6 polyubiquitination in cervical cancer cells (Preston et al., 2016). Ubiquitination initiates the degradation of the immunity protein, freeing the toxin to initiate cell death. **(C)** Taking advantage of the upregulation of specific proteases in cancer and virus-infected cells, a fusion toxin/immunity protein can be designed with a linker that is selectively cleaved by an upregulated protease (Chan et al., 2015; Yeo et al., 2016). Protease cleavage frees the toxin and results in cell death. **(D)** A DNA sequence complementary to an onco-miRNA is placed upstream to the immunity gene, and functions as an osPOM. When the toxin and immunity genes are transcribed, the oncomiRNA binds the complementary sequence and initiates degradation of the immunity mRNA. Thus, in cancer cells, transcription of the immunity gene is silenced while toxin transcription continues, leading to toxin-induced cell death (Turnbull et al., 2019; Houri et al., 2020).

Because TA and CDI systems are present in bacterial pathogens and because TA and CDI genes have no human homologs, TA pairs and CdiA-CT/CdiI complexes make attractive antibacterial drug targets (Williams and Hergenrother, 2012; Unterholzner et al., 2013; Chan et al., 2015). Antibacterial efforts with TA pairs have largely centered on direct or indirect activation of its toxin. Direct activation involves disrupting the toxin-antitoxin interface using small molecules (Figure 5A), while indirect toxin activation involves expedited degradation of the antitoxin (Figure 5B). For example, a peptide known as extracellular death factor (EDF) has been implicated in the activation of the MazF toxin from the *E. coli mazEF* TA system in the presence of its antitoxin, MazE (Williams and Hergenrother, 2012; Unterholzner et al., 2013). EDF binds to MazF preventing complex formation with MazE, resulting in enhanced MazF cytotoxic activity. The goal in antibacterial efforts would be to disrupt CdiA-CT/CdiI complex formation without loss of toxin function. Subsequently, perhaps the best strategy involves targeting the immunity protein surface rather than CdiA-CT. To test this strategy, a macrocyclic peptide was designed to prevent formation of the CdiA-CT/CdiI^TA271^ complex by replacing the CdiA-CT extended β-hairpin at the toxin/immunity interface (Figure 3A) (Morse et al., 2015). While the peptide was able to form a β-sheet with CdiI, it was not able to outcompete CdiA-CT. Optimization of the peptide could potentially yield the desired effect, however, the experiment acts as proof of principle for the use of such peptides to fulfill CdiA-CT interactions at the CdiA-CT/CdiI interface.

An indirect route to activate the toxin is to remove the antitoxin from the cell either by targeted degradation or downregulation (Unterholzner et al., 2013; Chan et al., 2015). In TA systems—where the antitoxin is more susceptible to degradation than the toxin—upregulation of certain proteases could deplete the cellular antitoxin pool and result in toxin activation. In CDI, where the immunity protein is not targeted by specific proteases, this is likely not a good antibacterial avenue. However, preventing the transcription or translation of the immunity protein through antisense molecules is an avenue worth considering.

TA systems have also been applied to develop anti-viral and anti-cancer therapies. The principle is to create a TA system such that the toxin is only activated in virus-infected or cancer cells. Antiviral applications to date have centered on MazF, a well characterized endoribonuclease TA toxin mentioned above (Unterholzner et al., 2013; Chan et al., 2015; Yeo et al., 2016). In experiments targeting human immunodeficiency virus (HIV), *mazF* was placed under the control of the HIV-1 TAR promoter in CD4^+^ cells. When these cells were infected with HIV, the infection resulted in the expression of MazF, which cleaved viral mRNA preventing HIV replication. In experiments targeting Hepatitis C virus (HCV), a fusion protein was designed bearing an HCV serine protease cleavage site between MazF and part of its antitoxin MazE (Figure 5C). When cells bearing this fusion protein are infected with HCV, the fusion protein is cleaved, and the infected cells are destroyed due to MazF toxicity. As viruses frequently encode highly specific proteases involved in their pathogenicity, this experimental methodology could be used to target a variety of viruses. These experiments are excellent proof-of-principle for how TA toxins could be employed in anti-viral therapies.

Anti-cancer applications to date involve the design of TA pairs where the antitoxin is specifically degraded in cancer cells freeing the toxin to eradicate cancer cells (Preston et al., 2016; Turnbull et al., 2019; Houri et al., 2020). For example, in human papilloma virus (HR-HPV) induced cervical cancer cells, oncoprotein E6 binds and induces the polyubiquitination of specific target proteins, resulting in target protein degradation. A TA fusion protein—wherein the toxin and antitoxin were fused together with an oncogene-specific protein output modifier (osPOM)—was generated such that the osPOM is polyubiquitinated by oncoprotein E6 in cancer cells, resulting in proteasomal degradation of the N-terminal polyubiquitinated osPOM and antitoxin domain, and subsequent toxin activation (Figure 5B) (Preston et al., 2016). Another approach takes advantage of microRNA-driven oncogenic stress, where miRNA (noncoding RNA) base pairs with complementary target sites (miRts) to inhibit translation or induce degradation of target mRNAs. Here, a cancer cell specific miRt is encoded downstream of the antitoxin gene, resulting in cancer cell specific silencing of the antitoxin resulting in toxin activation (Figure 5D) (Turnbull et al., 2019; Houri et al., 2020). This strategy has been utilized by two different TA pairs along with two different miRNA/miRt sequences indicating that this approach could be applied to target specific cancer cells utilizing a variety of different TA systems.

CDI toxins could also be employed in anti-viral or anti-cancer therapeutics. As TA toxins and other bacterial toxins can kill human cancer cells, CDI toxins should also have this capability. CdiA-CT/CdiI fusions could be generated with viral protease cut sites that trigger release of CdiA-CT from its immunity protein (Figure 5C). As most CdiA-CT toxins have RNase-type activities, these CdiA-CT proteins are ideal for targeting viral RNA. To successfully release CdiA-CT from its cognate CdiI, the high affinity interaction between CdiA-CT and CdiI must be overcome. Previous experiments have already been successful at dramatically reducing the toxin/immunity affinity, where the substitution of nine CdiA-CT^TA271^ residues at the CdiA-CT/CdiI^TA271^ interface resulted in a ∼2,500 × decrease in affinity (Morse et al., 2015). Similarly, targeted degradation or silencing of CdiI using cancer proteasomal degradation signaling or miRt incorporation is also an attractive avenue to explore (Figure 5D).

To conclude, CDI toxins and immunity proteins represent a new tool for therapeutic and biotechnological advancement. Experiments that have been performed with TA systems are excellent starting points to explore the usefulness of CDI as a manipulatable tool. Vast groundwork has been made in the characterization of CDI toxin/immunity pairs; the structural knowledge discussed in this review will empower novel applications of these proteins. Lastly, CDI comprises more than toxin and immunity proteins; CDI is a toxin delivery system that can selectively target specific bacterial strains. Thus, work to harness and manipulate the CDI mechanisms of CdiA-CT delivery could be a powerful tool to selectively deliver protein cargo to or to arm commensal bacteria against bacterial pathogens.

## Concluding Remarks

CDI is one of several microbial secretion systems that deploy antibacterial effectors to ensure bacterial fitness and survival. CDI has evolved to recognize and attack competing bacterial species to gain a competitive advantage. In this review we discussed the highly specific mechanisms CDI has acquired to deliver toxins into the target-cell cytosol, including recognition of different target-cell outer membrane proteins or polysaccharides and inner membrane proteins. Further, we have highlighted the structural and functional diversity of CDI toxins along with their cognate immunity proteins, including a discussion of the few known toxins that require activation by target-cell house-keeping proteins. These target-cell accessory proteins also reinforce toxin diversity as even when the same effector is utilized—like with the three toxins that require EF-Tu—toxin-activation is dissimilar. The CDI field is still in its infancy, and we predict that many other functionally diverse toxins and target-cell toxin-activating accessory proteins will be discovered. Thus, CDI offers a rich array of untapped biochemistry both in target-cell recognition, delivery, and cytotoxic proteins that could be leveraged to develop novel antibacterial biomedical therapies.
